# Spatial proteomics identifies JAKi as treatment for a lethal skin disease

**DOI:** 10.1038/s41586-024-08061-0

**Published:** 2024-10-16

**Authors:** Thierry M. Nordmann, Holly Anderton, Akito Hasegawa, Lisa Schweizer, Peng Zhang, Pia-Charlotte Stadler, Ankit Sinha, Andreas Metousis, Florian A. Rosenberger, Maximilian Zwiebel, Takashi K. Satoh, Florian Anzengruber, Maximilian T. Strauss, Maria C. Tanzer, Yuki Saito, Ting Gong, Marvin Thielert, Haruna Kimura, Natasha Silke, Edwin H. Rodriguez, Gaetana Restivo, Hong Ha Nguyen, Annette Gross, Laurence Feldmeyer, Lukas Joerg, Mitchell P. Levesque, Peter J. Murray, Saskia Ingen-Housz-Oro, Andreas Mund, Riichiro Abe, John Silke, Chao Ji, Lars E. French, Matthias Mann

**Affiliations:** 1 Department of Proteomics and Signal Transduction; Max Planck Institute of Biochemistry, Martinsried, Germany; 2 Department of Dermatology, University Hospital Zurich, University of Zurich, Zurich, Switzerland; 3 Department of Dermatology and Allergy, University Hospital, Ludwig Maximilian University (LMU) Munich, Munich, Germany; 4 Inflammation division, Walter and Eliza Hall Institute of Medical Research, Melbourne, Victoria Australia; 5 Department of Medical Biology, University of Melbourne, Parkville, Victoria Australia; 6 Division of Dermatology, Niigata University Graduate School of Medical and Dental Sciences, Niigata, Japan; 7 Department of Dermatology, The First Affiliated Hospital of Fujian Medical University, Fuzhou, China; 8 Department of Internal Medicine, Division of Dermatology, Cantonal Hospital Graubuenden, Chur, Switzerland; 9 Proteomics Program, Novo Nordisk Foundation Center for Protein Research, University of Copenhagen, Faculty of Health and Medical Sciences, Copenhagen, Denmark; 10 Advanced Technology and Biology division, Walter and Eliza Hall Institute of Medical Research, Melbourne, Victoria Australia; 11 Immunoregulation Research Group, Max Planck Institute of Biochemistry, Martinsried, Germany; 12 Department of Dermatology, Inselspital, Bern University Hospital, University of Bern, Bern, Switzerland; 13 Division of Allergology and Clinical Immunology, Department of Pneumology, Allergology and Clinical Immunology, Inselspital, Bern University Hospital, University of Bern, Bern, Switzerland; 14 Dermatology Department, AP-HP, Henri Mondor Hospital, Créteil, France; 15 Key Laboratory of Skin Cancer of Fujian Higher Education Institutions, Fujian Medical University, Fuzhou, China; 16 Dr. Philip Frost Department of Dermatology and Cutaneous Surgery, University of Miami Miller School of Medicine, Miami, FL USA

**Keywords:** Proteomics, Translational research, Diseases

## Abstract

Toxic epidermal necrolysis (TEN) is a fatal drug-induced skin reaction triggered by common medications and is an emerging public health issue^1–3^. Patients with TEN undergo severe and sudden epidermal detachment caused by keratinocyte cell death. Although molecular mechanisms that drive keratinocyte cell death have been proposed, the main drivers remain unknown, and there is no effective therapy for TEN^4–6^. Here, to systematically map molecular changes that are associated with TEN and identify potential druggable targets, we utilized deep visual proteomics, which provides single-cell-based, cell-type-resolution proteomics^7,8^. We analysed formalin-fixed, paraffin-embedded archived skin tissue biopsies of three types of cutaneous drug reactions with varying severity and quantified more than 5,000 proteins in keratinocytes and skin-infiltrating immune cells. This revealed a marked enrichment of type I and type II interferon signatures in the immune cell and keratinocyte compartment of patients with TEN, as well as phosphorylated STAT1 activation. Targeted inhibition with the pan-JAK inhibitor tofacitinib in vitro reduced keratinocyte-directed cytotoxicity. In vivo oral administration of tofacitinib, baricitinib or the JAK1-specific inhibitors abrocitinib or upadacitinib ameliorated clinical and histological disease severity in two distinct mouse models of TEN. Crucially, treatment with JAK inhibitors (JAKi) was safe and associated with rapid cutaneous re-epithelialization and recovery in seven patients with TEN. This study uncovers the JAK/STAT and interferon signalling pathways as key pathogenic drivers of TEN and demonstrates the potential of targeted JAKi as a curative therapy.

## Abstract (web summary)

Cell-type-resolved spatial proteomics of the skin from patients with toxic epidermal necrolysis reveals that it is driven by JAK/STAT signaling, leading to successful treatment of this potentially fatal condition in patients using JAK inhibitors.

## Main

The skin is the organ that is most frequently affected by adverse drug reactions, and nearly 2% of such reactions are severe and life-threatening^9^. The spectrum of cutaneous adverse drug reactions (CADRs) ranges from self-resolving maculopapular rash (MPR) to rare and life-threatening conditions, such as drug reaction with eosinophilia and systemic symptoms (DRESS), Stevens–Johnson syndrome (SJS) and TEN^5^. TEN is characterized by fulminant epidermal detachment exceeding 30% of the body surface area, whereas its milder form SJS–TEN overlap disease affects 10−30% of skin surface area. TEN is fatal in one-third of all cases. Despite diverse proposed pathogenic mechanisms^10–24^, the main drivers of cytotoxicity remain unknown and consensus therapy of TEN remains primarily supportive care^25^.

Spatial omics has recently emerged as a powerful strategy to obtain a global, molecular view of intact tissue samples^26,27^. When applied at single-cell resolution, these methods can profile different cell types within their native environment. Although a variety of such technologies exist, translation into direct clinical benefit remains largely aspirational. Deep visual proteomics (DVP) provides spatial proteomics data of individual cell types from formalin-fixed paraffin-embedded (FFPE) archived biopsies. DVP combines high content imaging, artificial intelligence-guided cell segmentation and classification and laser microdissection of individual target cells coupled with ultra-sensitive mass spectrometry-based proteomics^7^. Here, we utilized DVP to study mechanisms of cutaneous drug reactions, investigate molecular features in the different CADRs, and functionally validate targeted small-molecule inhibitors for rapid therapeutic intervention in TEN, the most severe CADR.

## DVP workflow

**Fig. 1:**
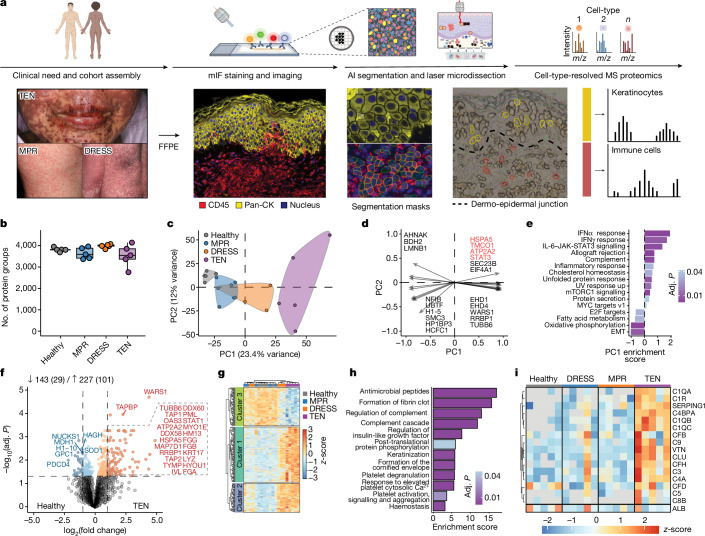
DVP workflow and cell-type-specific proteome of keratinocytes in cutaneous drug reactions. **a**, Top, schematic of the DVP workflow for cell-type-resolved proteomics in CADRs (*n* = 21; 5 each for DRESS, MPR and healthy, and 6 for TEN). Bottom, representative images of each step, including excised keratinocytes (outlined in yellow in the image on the far right) and immune cells (outlined in red in the far right image) from FFPE tissue sections. Partially created with Biorender.com. AI, artificial intelligence. mIF, multicolour immunofluorescence; MS, mass spectrometry; pan-CK, pan-cytokeratin. **b**, Number of proteins identified in keratinocytes across cohorts. *n* = 5 individuals per cohort. **c**–**e**, Principal components analysis (**c**), main drivers (loadings) of PC1–PC2 separation (**d**) and gene set enrichment analysis (**e**) (GSEA) (MSigDB Hallmark) of PC1. Adj. *P*, adjusted *P* value; EMT, epithelial–mesenchymal transition. **f**, Differential expression of proteins in TEN and healthy keratinocytes. Coloured dots indicate significant DEPs, the dashed vertical line indicates log_2_-transformed fold change of ±1. Numbers indicate the total number of DEPs in each direction. *n* = 5 individuals per cohort. **g**, Hierarchical clustering of significant proteins by ANOVA. Colour indicates normalized intensity (*z*-score). *n* = 5 individuals per cohort. **h**, Overrepresentation analysis (Reactome) of ANOVA cluster 1, ordered by enrichment score. Colour indicates degree of significance. **i**, Semi-supervised heat map of complement factors (and albumin to exclude blood contamination) in the indicated conditions. Colour indicates normalized values (*z*-score). *n* = 5 individuals per cohort. **f**, Unpaired two-sided *t*-test. **g**, One-way ANOVA with post hoc Tukey’s Honest significant difference (HSD). **e**–**h**, Benjamini–Hochberg correction for multiple comparisons (false discovery rate (FDR) < 0.05). In box plots the centre is the media, box edges delineate the 25th to 75th centiles and whiskers extend to furthest points within 1.5× the interquartile range.

**Extended Data Fig. 1:**
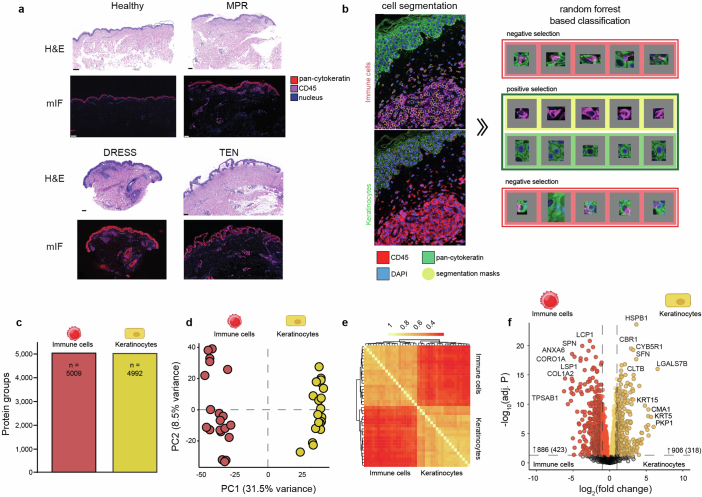
Deep Visual Proteomics workflow. **a**, Side-by-side H&E and multicolor immunofluorescence (mIF) of 3 μm FFPE tissue sections across all cohorts, representative images shown. Scale bar, 200 µm. **b**, Machine learning based image segmentation was followed by random-forest based classification to ensure no overlapping contours (‘negative selection’) were microdissected, yielding pure and cell-type specific contours (‘positive selection’). **c,d**, Identification of unique protein groups (c) and principal component analysis (d) across all patients in the indicated cell type. Each dot represents a patient, color represents the cell type. **e,f**, Pearson correlation (e) and differentially expressed proteins (DEP, f) quantified across all patients in the indicated cell type. Colored dots are significant DEPs, dashed vertical line indicate log2 fold change of ≤ 1 or ≥ 1. Unpaired two-sided t-test with Benjamini-Hochberg correction for multiple comparisons (FDR < 0.05). **a-f**, n = 21 individuals (5 each DRESS/MPR/Healthy, 6 for TEN). TEN, Toxic Epidermal Necrolysis; MPR, Maculopapular Rash; DRESS, Drug Reaction with Eosinophilia and Systemic Symptoms.

We performed DVP on a retrospective cohort of lesional FFPE skin biopsies from patients with mild (MPR) and severe (TEN or DRESS) CADRs, as well as healthy individuals (Fig. 1a). We stained FFPE tissue sections for CD45^+^ immune cells and pan-cytokeratin^+^ keratinocytes, followed by machine learning-based cell segmentation (Extended Data Fig. 1a,b). For each individual, contours per cell type were laser microdissected and combined separately to maintain patient and cell-type specificity, prior to peptide extraction and acquisition of mass spectrometry data (Fig. 1a). This identified around 5,000 unique proteins in each cell type across all participants, with distinct profiles for keratinocytes and immune cells (Extended Data Fig. 1c–f).

## Proteome of lesional keratinocytes

**Extended Data Fig. 2:**
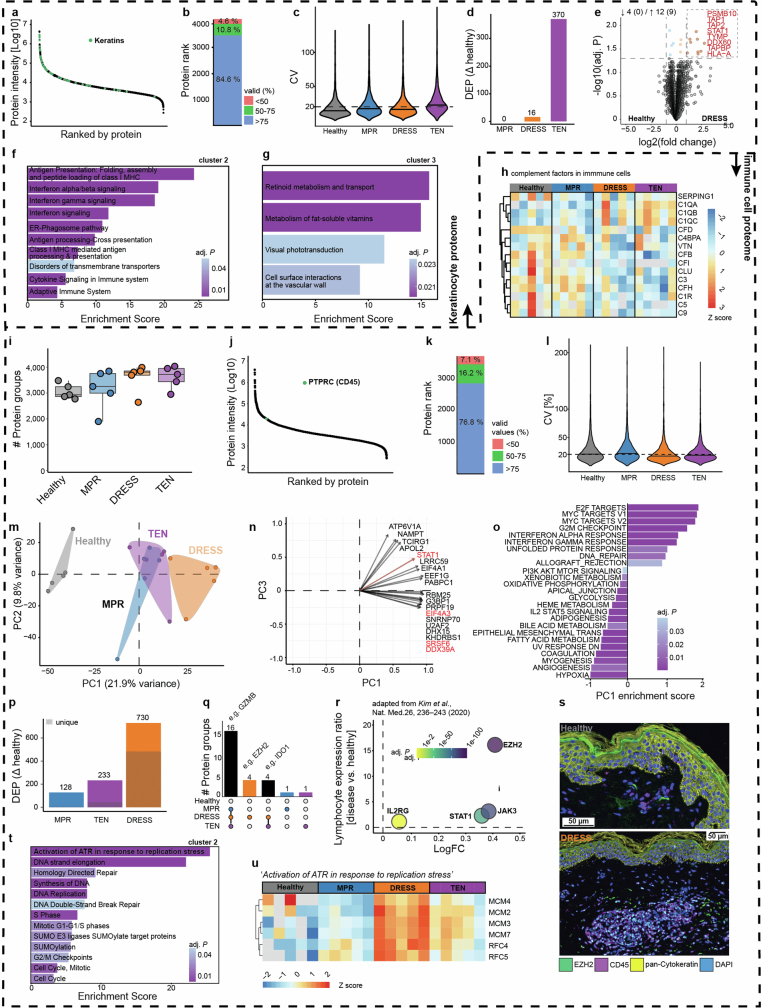
Keratinocyte and immune cell proteome. **a-c**, Rank plot of median protein intensity (a), data completeness per protein (b) and coefficient of variation (CV) per cohort (c) of the keratinocyte proteome. Keratins are highlighted in green (a). **d**, Number of differentially expressed proteins (DEP) in keratinocytes of MPR/DRESS/TEN compared to healthy. **e**, DEPs in keratinocytes of DRESS versus healthy. Colored dots are significant DEPs, dashed vertical line indicate log2 fold change of ≤ 1 or ≥ 1. Numbers indicate DEPs in each direction. **f, g**, Overrepresentation analysis (reactome) of ANOVA cluster 2 and 3 (shown in Fig. 1g), ordered by enrichment score. Color indicates degree of significance. **h**, Semisupervised heatmap of complement factors in immune cells of the indicated conditions. Color indicates normalized intensity levels (z score). **i**, Number of proteins identified in immune cells across cohorts. **j-l**, Rank plot of median protein intensity (j), data completeness per protein (k) and coefficient of variation (CV) per cohort (l) of the immune cell proteome. Ptprc is highlighted in green (j). **m-o**, Principal component analysis (m), top 20 proteins (loadings) contributing to PC1/ 3 (n) and PC1 gene set enrichment analysis (MSigDB Hallmark) of immune cells, ordered according to enrichment score (o). **p**, DEPs in the indicated conditions compared to healthy. Shaded area within columns indicates the proportion of non-overlapping differentially regulated proteins. **q**, Upset plot of uniquely identified proteins specific to each of the indicated conditions. **r**, Source Data from Kim, D., Kobayashi, T., Voisin, B. et al. Targeted therapy guided by single-cell transcriptomic analysis in drug-induced hypersensitivity syndrome: a case report. Nat Med 26, 236–243 (2020). Scatter plot of EZH2 and previously identified markers (STAT1, JAK3, IL2RG) in DRESS showing fold change (x-axis) and the ratio of cell expression in diseased versus healthy samples (y-axis) in PBMC lymphocyte clusters of DRESS. **s**, Representative histology stained for EZH2, CD45 and pan-Cytokeratin in DRESS (n = 5) and healthy (n = 5) skin tissue sections. Representative images shown. **t,u**, Overrepresentation analysis (“Reactome”) of ANOVA cluster 2 (shown in Fig. 2g), ordered by enrichment score (t) and semisupervised heatmap of enriched proteins of “activation of ATR in response to replication stress” (u). Color indicates degree of statistical significance (t) and normalized intensities (u). **d,e,p** unpaired two-sided t-test. **d-g,p,t**, Benjamini-Hochberg correction for multiple comparisons (FDR < 0.05). **a-q,t-u**, n = 5 individuals per cohort (MPR, DRESS, TEN, healthy). Box plots: median (center), interquartile range (box, 25th-75th percentiles), whiskers (furthest points within 1.5 * interquartile range). TEN, Toxic Epidermal Necrolysis; MPR, Maculopapular Rash; DRESS, Drug Reaction with Eosinophilia and Systemic Symptoms.

To identify disease- and cell-type-specific molecular mechanisms, we first analysed the protein expression profiles of lesional keratinocytes from the CADRs (Fig. 1b and Extended Data Fig. 2a–c). Keratinocyte proteomes clustered according to the type of drug reaction, with principal component 1 (PC1) separating TEN from other CADRs and healthy individuals (Fig. 1c). PC1 was strongly associated with proteins involved in calcium homeostasis and cellular stress response (HSPA5, TMCO1 and ATP2A2; Fig. 1d). Of note, STAT3 was a main driver of separation, along with a dominant interferon signature (Fig. 1d,e). The number of differentially expressed proteins (DEPs) compared with cells from healthy individuals increased with the severity of the CADRs (Extended Data Fig. 2d). DRESS keratinocytes expressed more than double the amount of the major histocompatibility complex class I proteins TAP1, TAP2, TAPBP and HLA-A, suggesting that keratinocytes participate actively in culprit-drug-associated antigen presentation (Extended Data Fig. 2e). Among significantly regulated proteins in keratinocytes from patients with TEN, many were involved in antimicrobial response, such as lysozyme and clusterin (Fig. 1f). WARS1, one of the most potently upregulated proteins, is strongly induced by inflammatory signals and especially IFNγ^28^, and has not been associated with TEN. Superoxide dismutase 1 (SOD1) levels were halved in the TEN keratinocytes, potentially exacerbating the oxidative stress cascade that is known to upregulate keratinocyte expression of Fas ligand, a key cytolytic molecule that is involved in keratinocyte apoptosis and epidermal detachment in TEN^20,29^.

To identify protein signatures of keratinocytes that are uniquely associated with specific CADRs, we examined hierarchical clusters of DEPs, which showed a clear segregation for TEN among three distinct protein clusters (Fig. 1g). The TEN cluster 1 was enriched for terms involved in the regulation of complement activation and blood clotting (Fig. 1h and Extended Data Fig. 2f,g). In accordance, nearly all complement factors were uniquely upregulated in keratinocytes of TEN and their presence may reflect the degree of dying keratinocytes in TEN (Fig. 1i and Extended Data Fig. 2h). Thus, proteomes of keratinocytes derived from DRESS and TEN reveal distinct disease-associated protein expression signatures.

## Proteome of lesional immune cells

**Fig. 2:**
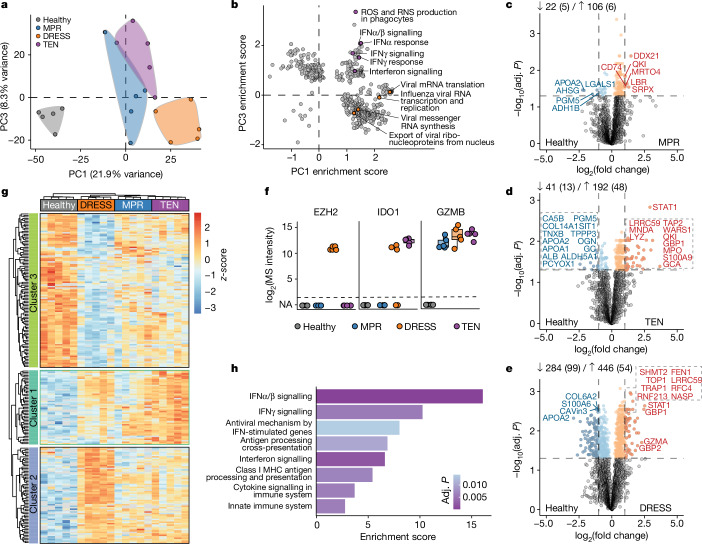
Cell-type-specific proteome of lesional immune cells. **a**, Principal component analysis. *n* = 5 individuals per cohort. **b**, Scatter plot of gene set enrichment scores (MsigDB Hallmark, Gene Ontology Biological Processes) present in both PC1 and PC3. Terms that include ‘viral’ or ‘interferon’ are highlighted. RNS, reactive nitrogen species; ROS, reactive oxygen species. **c**–**e**, DEPs between healthy and MPR (**c**), TEN (**d**) or DRESS (**e**) immune cells. Coloured dots indicate significant DEPs, dashed vertical lines indicate log_2_-transformed fold change of ±1. Numbers indicate the total number of DEPs in each direction. *n* = 5 individuals per cohort. **f**, Selected proteins with distinct intensities across cohorts. **g**, Hierarchical clustering of significant proteins by ANOVA. Colour indicates normalized intensities (*z*-score). *n* = 5 individuals per cohort. **h**, Overrepresentation analysis (Reactome) of ANOVA cluster 1, ordered by enrichment score. MHC, major histocompatibility complex. Colour indicates degree of significance. **c**–**e**, Unpaired two-sided *t*-test. **g**, One-way ANOVA with post hoc Tukey’s HSD. **b**–**e**,**g**,**h**, Benjamini–Hochberg correction for multiple comparisons (FDR < 0.05).

Cutaneous immune cell infiltration patterns differ markedly among CADRs^30^. To understand underlying molecular differences in immune cells, we assessed their proteomes (Extended Data Fig. 2i–l). They clustered by disease phenotype, but in contrast to keratinocytes, PC1 separated DRESS from other CADRs (Fig. 2a and Extended Data Fig. 2m). It was prominently associated with targets of E2F and MYC, indicating a highly proliferative state (Extended Data Fig. 2o). PC1 and PC3 together revealed unique features of DRESS and TEN. In DRESS, multiple viral pathway terms were enriched, consistent with viral reactivation in its pathogenesis (Fig. 2b). By contrast, TEN had a dominant interferon signature with STAT1 as a main driver (Fig. 2b and Extended Data Fig. 2n). DRESS resulted in the largest number of DEPs compared with healthy immune cells, followed by TEN and MPR (Fig. 2c–e and Extended Data Fig. 2p). Few proteins were unique to specific CADR subsets (Extended Data Fig. 2q). One such protein was histone methylase EZH2, which was detectable only in immune cells from patients with DRESS (Fig. 2f). This proteomic observation was confirmed by immunofluorescence and by a report of almost exclusive transcription in a patient with therapy-resistant DRESS^31^ (Extended Data Fig. 2r,s). Among proteins previously associated with specific CADRs, granzyme B has been described as a key mediator of cytotoxicity in TEN^24^, however, it was present in similar amounts in non-cytotoxic DRESS (Fig. 2f).

Unsupervised hierarchical clustering analysis on DEPs in immune cells across all cohorts revealed three visually distinct protein clusters with perfect disease alignment (Fig. 2g). Cluster 2, which predominantly represents DRESS, was enriched for DNA replication processes, highlighting the proliferative nature of infiltrating immune cells in this disease (Extended Data Fig. 2t). Its most prominent term (activation of ATR in response to replication stress) was dominated by the DNA replication factors RFC and MCM, establishing a potential link to the viral pathogenesis of DRESS^32^ (Extended Data Fig. 2u). Notably, cluster 1 contained proteins that are predominantly associated with type I and II interferon signalling (Fig. 2h). These proteins were markedly overexpressed in TEN, but showed only slightly increased expression in MPR and DRESS (Fig. 2g).

## Prominent macrophage involvement in TEN

**Fig. 3:**
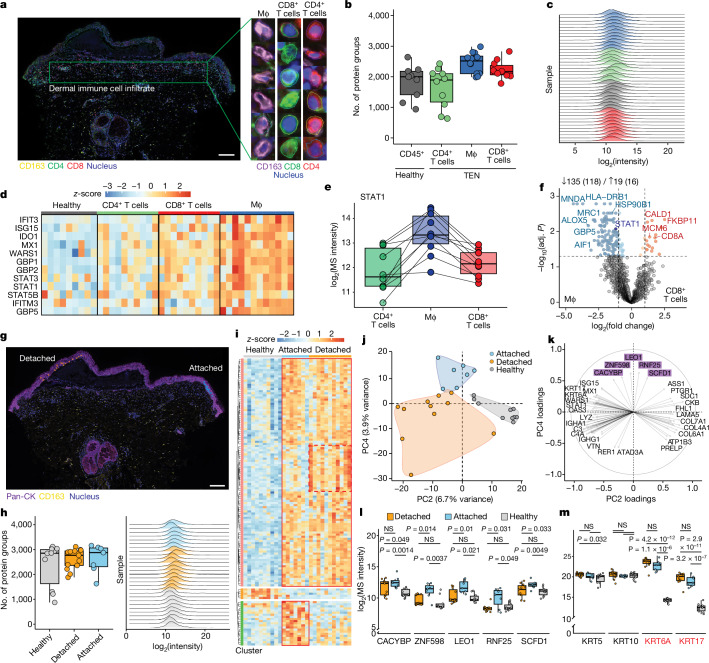
Spatial proteomic profiling of immune cell subtypes in TEN. **a**, Left, immunofluorescence image of TEN with segmented CD163^+^ macrophages, CD4^+^ and CD8^+^ T cells. Right, representative single-cell images and segmentation contours. Mϕ, macrophage. Scale bar, 200 μm. **b**,**c**, Number of identified proteins (**b**) and their intensity (**c**) across all samples, colour-coded by cell type. **d**, Heat map of interferon pathway proteins across samples grouped by cell type. Colour indicates normalized intensities (*z*-score). **e**, STAT1 intensity values across immune cell subtypes. Lines connect measurements from the same patient. **f**, DEPs between indicated cell types. Coloured dots represent significant DEPs, dashed lines indicate log_2_-transformed fold change of ±1. Numbers indicate the total number of DEPs in each direction. **g**, Immunofluorescence of a TEN sample with segmented keratinocytes from detached (orange; blister roof) and attached (blue; adjacent to blister) regions. Scale bar, 200 μm. **h**, Number of proteins identified in keratinocytes (left) and intensity distribution (right) across samples. **i**, Semi-supervised hierarchical clustering of significant proteins by ANOVA with fold change greater than 1. Colour indicates normalized intensities (*z*-score). Borders of visually distinct subclusters are outlined in red. **j**,**k**, Principal component analysis (**j**) and main drivers of PC2 and PC4 (**k**). **l**,**m**, Proteins of interest that vary between detached versus attached cells (**l**; highlighted in **k**) and across cohorts (**m**). Alarmins are labelled in red. **b**–**d**, *n* = 12 macrophage, 10 CD4^+^ cell, 10 CD8^+^ cell and 9 healthy samples from *n* = 12 disease-affected and 7 healthy individuals. **e**,**f**, paired samples for each cell type in *n* = 10 individuals. **h**–**m**, *n* = 7 attached, 11 detached and 9 healthy samples from *n* = 11 disease-affected and 7 healthy individuals. **a**,**g**, Immunofluorescence staining was performed on all samples, representative images are shown. **f**, Paired two-sided *t*-test. **i**, One-way ANOVA with post hoc Tukey’s HSD. **f**,**i**, Benjamini–Hochberg correction for multiple comparisons (FDR < 0.05). NS, not significant.

Although they indicated a role for the interferon pathway in TEN, our experiments did not identify the specific immune cell subtypes that contributed to this response. To this end, we harnessed a multiplexed data-independent acquisition (mDIA) workflow with a reference channel and the novel Astral mass analyser for the DVP workflow^33,34^. This enabled proteomic investigation of CD163^+^ macrophages, CD4^+^ T helper cells and CD8^+^ cytotoxic T cells infiltrating the skin of patients with TEN and SJS–TEN overlap, despite their extreme sparsity (Fig. 3a). From only 20 cell equivalents per immune cell type and participant, we covered a median of 2,104 and a total of 5,302 proteins (Fig. 3b,c). Macrophages had the highest expression of proteins related to the interferon pathway, potentially reflecting the higher expression of IFNγ receptor (Fig. 3d). In particular, the amount of the mediator of interferon signalling STAT1 was consistently higher in macrophages than in CD4^+^ and CD8^+^ T cells from the same individuals (Fig. 3e). Compared with cytotoxic T cells, macrophages showed significant enrichment for myeloid-specific lineage markers (MNDA and MRC1) and interferon-driven proteins (STAT1 and GBP5; Fig. 3f).

## Spatial proteome of epidermal detachment

**Extended Data Fig. 3:**
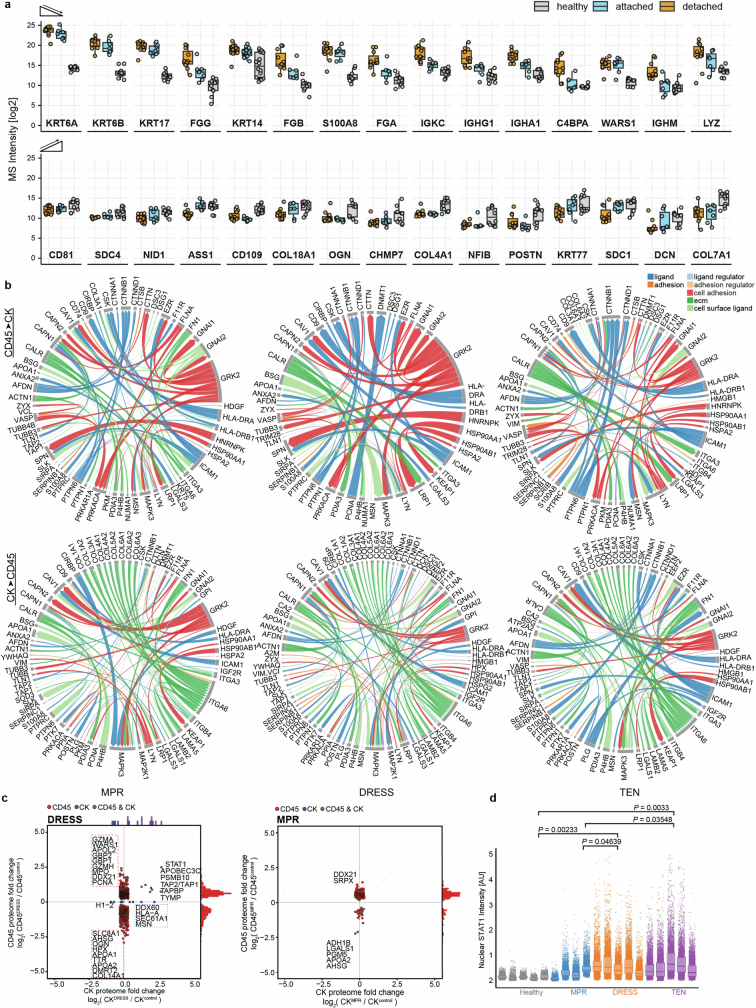
**A**, Top 15 proteins that significantly follow a linear relationship from healthy to attached to detached keratinocytes. The proteins are displayed in order of highest MS intensity values in detached keratinocytes (top panel) and healthy keratinocytes (bottom panel). Colors denote sample origin. Box plots: median (center), interquartile range (box, 25th-75th percentiles), whiskers (furthest points within 1.5 * interquartile range). **b**, Circos plots to visualize ligand-receptor interactions between keratinocytes (CK) and immune cells (CD45) across the cutaneous drug reactions (MPR, DRESS, TEN), normalized to healthy. In the bottom panels (CK > CD45) immune cells are the recipient, while in the top panels (CD45 > CK) keratinocytes are the recipient. Arrows indicate interaction direction, colors the different functional categories as indicated, and width of arrow the difference in average expression levels. **c**, Differentially expressed proteins in keratinocytes (x-axis) and immune cells (y-axis) of DRESS (left) or MPR (right) compared to healthy. Each dot represents a protein, color indicates cell type. n = 5 individuals per cohort and cell type. Histograms above and to the right display the distribution of fold changes along the respective cell type. **d**. Nuclear Stat1 intensity per cell (shown in Fig. 4e), normalized to its nuclear stain intensity. Each dot represents a single cell measurement. n = 5000 cells per individual, n = 5 individuals each for TEN, DRESS, Healthy, 4 for MPR. Box plots: median (center), interquartile range (box, 25th-75th percentiles). Unpaired two-sided t-test on mean normalized Stat1 values. TEN, Toxic Epidermal Necrolysis; MPR, Maculopapular Rash; DRESS, Drug Reaction with Eosinophilia and Systemic Symptoms.

We next investigated spatial differences between detached (blister roof) and attached (blister side) keratinocytes from within the same biopsy from only 20 cells per type and patient (Fig. 3g,h). Among three distinct clusters, the largest comprised the complement system and inflammation (Fig. 3i). These proteins were upregulated in both attached and detached keratinocytes of TEN, indicating that the inflammatory pathways driving TEN are already active in keratinocytes adjacent to blistered skin. Several proteins followed a linear relationship from healthy to attached to detached keratinocytes (Extended Data Fig. 3a). Proteomic differences between attached and detached keratinocytes were subtle, but sufficient to separate them according to spatial location and away from healthy keratinocytes (Fig. 3j). This separation was again driven by proteins of the complement and inflammation cascade (including MX1, C3, KRT6 and KRT16, among others; Fig. 3k–m). Furthermore, five proteins were associated with attached versus detached cells and may represent early proteomic changes before detachment (Fig. 3k–m).

## The JAK/STAT pathway is highly active in TEN

**Fig. 4:**
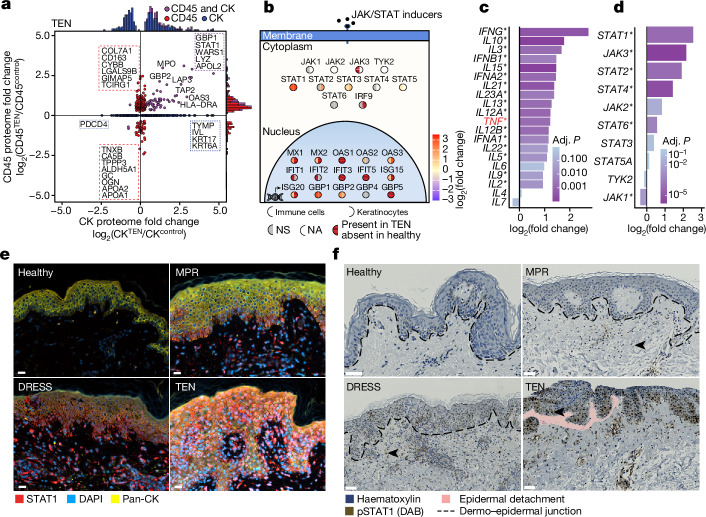
The JAK/STAT pathway is potently activated in TEN. **a**, DEPs in keratinocytes (CK) and immune (CD45) cells in patients with TEN compared with healthy participants. Each dot represents a protein and colour indicates cell type. Histograms above and to the right display the distribution of fold changes for the indicated cell type. *n* = 5 individuals per cohort and cell type. **b**, JAK/STAT pathway proteins in immune cells (left semicircle) and keratinocytes (right semicircle). Colour represents fold change in TEN compared with healthy donors; red with horizontal black lines indicates proteins identified in TEN only. *n* = 5 individuals per cohort and cell type. NA, data not available. **c**,**d**, Differentially expressed mRNA transcripts in TEN compared with healthy tissue for selected cytokines (**c**) and components of the JAK/STAT pathway (**d**). Asterisks indicate significant differences between groups. *n* = 10 individuals per cohort. TNF is highlighted. **e**,**f**, Representative images of STAT1 and pan-cytokeratin (**e**) and phosphorylated STAT1 (pSTAT1) (**f**) staining in tissue sections across cohorts. *n* = 5 individuals per cohort, representative images shown. Scale bars: 20 μm (**e**), 50 μm (**f**). **a**–**d**, Unpaired two-sided *t*-test. **a**,**c**,**d**, Benjamini–Hochberg correction for multiple comparisons (FDR < 0.05).

**Extended Data Fig. 4:**
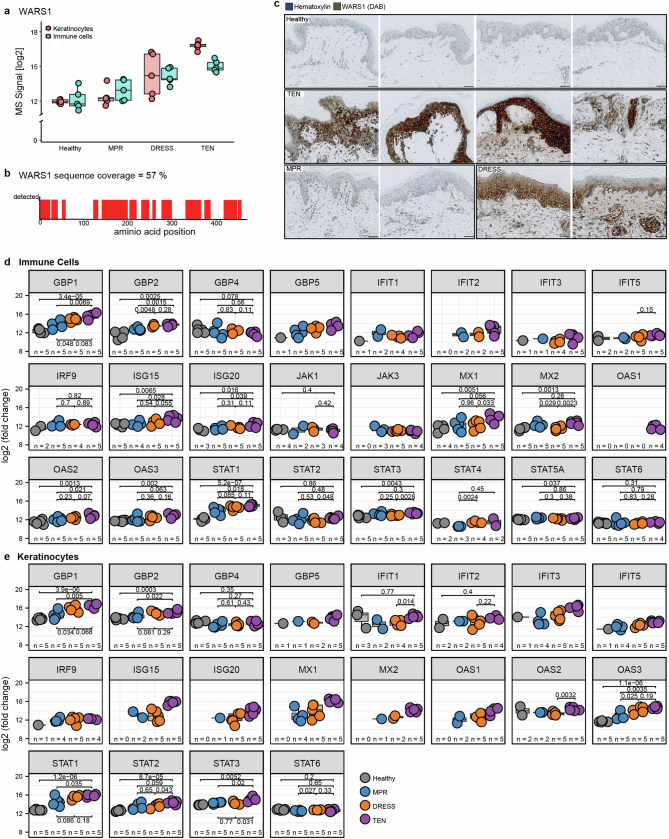
WARS1 expression in cutaneous drug reactions. **a**. Protein expression level of WARS1 across all cohorts and cell types as indicated. **b**, Identified amino acid sequence and sequence coverage of WARS1 using mass-spectrometry across all samples. **c**, Histology of WARS1 across cohorts as indicated. Scale bar = 50 µm. **d,e**, Differential expression analysis of proteins depicted in Fig. 5b for immune cells (c) and keratinocytes (d). Two-sided t-test. Valid values per protein and cohort as depicted. Box plots: median (center), interquartile range (box, 25th-75th percentiles), whiskers (furthest points within 1.5 * interquartile range). TEN, Toxic Epidermal Necrolysis; MPR, Maculopapular Rash; DRESS, Drug Reaction with Eosinophilia and Systemic Symptoms.

Severe inflammatory disorders are characterized by altered cellular cross-talk and feedback loops that exacerbate the immune response^35^; we investigated this using the cell-type-resolved proteome (Extended Data Fig. 3b). To further unravel such disease-specific feedback loops, we hypothesized that proteins that were strongly regulated in both keratinocytes and immune cells would identify therapeutically relevant pathways. Six such proteins (WARS1, STAT1, S100A9, LYZ, GBP1 and APOL2) were upregulated at least fourfold in keratinocytes and immune cells of TEN compared with healthy controls (Fig. 4a). We confirmed the proteomic findings of WARS1 using immunohistochemistry (Extended Data Fig. 4a–c). By contrast, DRESS had only one (STAT1) and MPR had no such overlapping proteins (Extended Data Fig. 3c). Notably, all six of these proteins are in the interferon pathway, responsible for signal transduction (via STAT1) or triggered by it^36–40^. Interferons signal through the JAK/STAT pathway; we therefore examined molecules in this pathway (Fig. 4b and Extended Data Fig. 4d,e). STAT1 and STAT3 were upregulated in both immune cells and keratinocytes, whereas STAT2 was upregulated only in keratinocytes, and STAT5 was upregulated in immune cells. Most interferon-stimulated gene (ISG) products were either potently upregulated, illustrated by a ninefold increase of GBP1, or only detectable in TEN—for example, GBP5.

**Extended Data Fig. 5:**
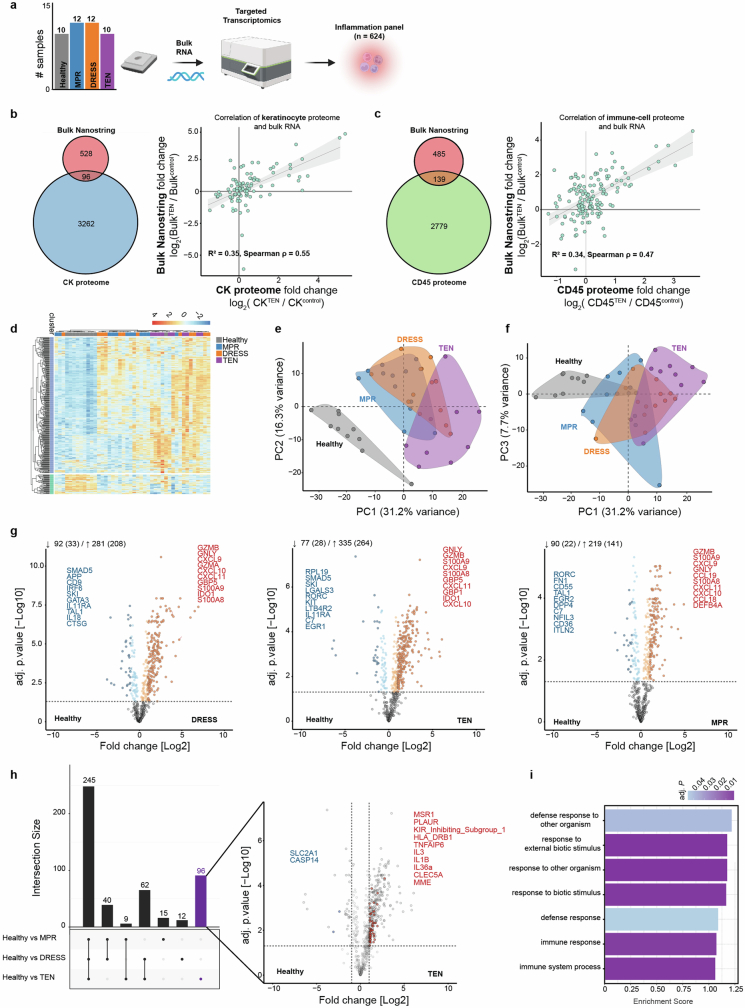
Targeted transcriptomics of bulk tissue sections. **a**, Schematic representation of the targeted transcriptomic analysis performed on bulk FFPE scrolls using the nCounter Inflammation panel. Number of measured transcripts as indicated (n = 624). **b-c**, Venn diagram of identified transcripts detected by targeted transcriptomics of bulk tissue (top) and proteins identified using DVP (bottom) of keratinocytes (b) or immune cells (c). Correlation analysis metrics for overlapping proteins / transcripts as indicated. **d**, Hierarchical clustering of ANOVA significant proteins. Color indicates normalized intensities (z score). **e,f**, Principal Component analysis (PCA) of transcriptome from bulk tissue sections across all cohorts as indicated. **g**, Differentially expressed transcripts between healthy and DRESS (left), TEN (middle) or MPR (right). Colored dots are significant transcripts, and the top ten in each direction are shown. **h,i**, Upset plot of differentially regulated transcripts across all conditions compared to healthy (h). Those uniquely regulated in TEN are color-marked in the original volcano plot (right), followed by overrepresentation analysis (GOBP, i). **a-i**, n = 44 individuals (10 each for Healthy, TEN and 12 for DRESS and MPR). **d**, One-way ANOVA with post-hoc Tukey HSD. **g,h**, unpaired two-sided t-test. **d, g-i**, Benjamini-Hochberg correction for multiple comparisons (FDR < 0.05). TEN, Toxic Epidermal Necrolysis; MPR, Maculopapular Rash; DRESS, Drug Reaction with Eosinophilia and Systemic Symptoms.

**Extended Data Fig. 6:**
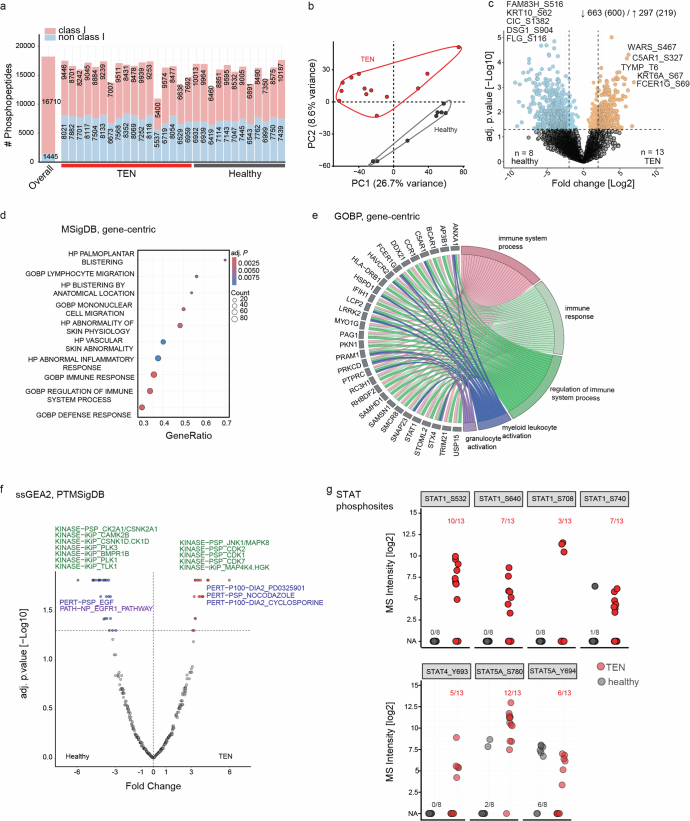
Phosphoproteomic analysis of TEN. **a**, Number of Class I phosphosites (localization probability > 75%; otherwise, non-class I) identified in TEN and Healthy per sample and overall. **b**, Principal Component Analysis (PCA). **c**, Differentially regulated phosphosites between TEN and healthy. Colored dots are significant, dashed vertical line indicate log2 fold change of ≤ 1 or ≥ 1. **d-e**, Gene-centric enrichment analysis shown as dotplot (MSigDB, d) or chord diagram of top five terms and related proteins (GOBP, e) of upregulated phosphosites in TEN. Size of dots correlate to the number of mapped proteins per term and color represents degree of statistical significance (d). **f**, Site-specific enrichment analysis (ssGEA2, PTMSigDB) of upregulated phosphosites in TEN. Points are color-coded according to the origin of the signature set (kinase-substrate interaction = green, perturbation = blue, molecular signaling pathway = purple). **g**, Number of identified STAT1/4/5 phosphosites in healthy and control samples, with numerical annotations indicating the identified phosphosites per group. **c-f**, Benjamini-Hochberg correction for multiple comparisons (FDR < 0.05). **a-g**, n = 8 (healthy) and 13 (TEN). TEN, Toxic Epidermal Necrolysis.

Cytokines activate the JAK/STAT pathway; to study the activation of this pathway, we utilized targeted transcriptomics in an expanded cohort of patients with CADR (Extended Data Fig. 5a–c). Among the genes encoding cytokines that are known to signal through the JAK/STAT pathway, *IFNG*(encoding IFNγ) expression was the most potently upregulated in TEN, exceeding the upregulation of genes such as *TNF*, which is already known to be involved in TEN^41^ (Fig. 4c). Whereas targeted transcriptomics was not able to separate between the different CADRs, specific interrogation of JAKs and STATs confirmed broad upregulation of these molecules in TEN and SJS–TEN overlap (Fig. 4d and Extended Data Fig. 5d–i). Antibody-based visualization of STAT1 in FFPE tissue sections further confirmed that it was significantly upregulated and localized to the nucleus in keratinocytes and other cell types in TEN (Fig. 4e and Extended Data Fig. 3d). Notably, the degree of single-cell nuclear STAT1 upregulation in immune cells of TEN was similar to that in DRESS, in which therapeutic intervention with JAKi has proved successful, despite their differing histopathology, clinical presentation and disease course^31^. This upregulation of STAT1 suggests that severe CADRs share a common inflammatory pathway, potentially driven by T cell activation. To further validate STAT1 activation in TEN at the signalling level, we assessed the phosphorylation status of STAT1. Histological analysis revealed a profound degree of STAT1 phosphorylation in skin-infiltrating immune cells and keratinocytes of patients with TEN (Fig. 4f). We extended this observation using global phosphoproteome analysis, measuring more than 16,000 class I phosphosites in an independent cohort of TEN, which further confirmed our findings (Extended Data Fig. 6). Collectively, these results demonstrate that activation of the JAK/STAT pathway is a key driver of TEN.

## Targeting JAK/STAT in models of TEN

**Fig. 5:**
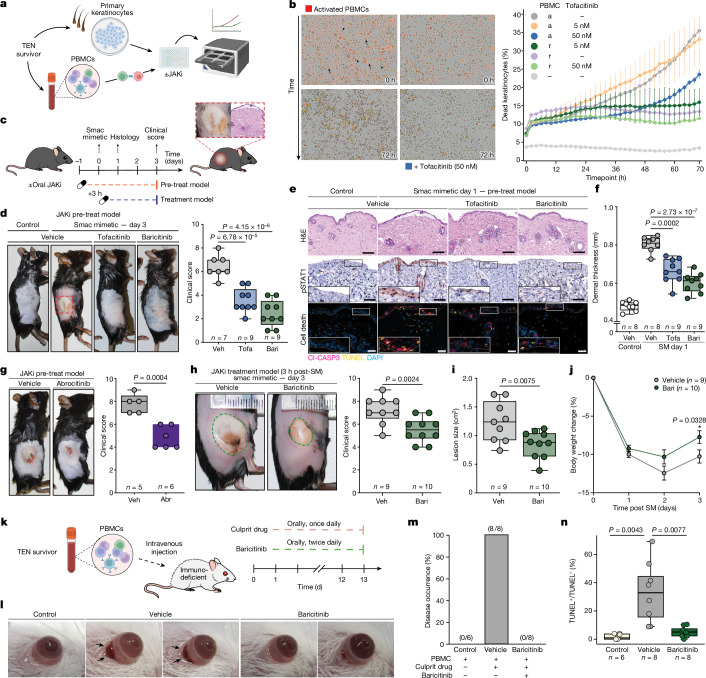
JAK/STAT inhibition reduces severity of TEN in vitro and in vivo. **a**,**b**, Schematic of live-cell imaging assay (**a**) with representative images of labelled keratinocytes (arrows) and unlabelled PBMCs (arrowhead) in co-culture for the indicated duration, with or without 50 nM tofacitinib to measure cytotoxicity over time (**b**). r, resting; a, activated. *n* = 6 biological replicates per condition, 4 field of views per well. Data are mean ± s.e.m. **c**, Schematic of oral JAKi treatment in the smac-mimetic-induced model of TEN. Oral JAKi (tofacitinib (tofa), 30 mg kg^−1^; baricitinib (bari), 10 mg kg^−1^; abrocitinib (abr), 20 mg kg^−1^) was started one day before (‘pre-treat model’) or 3 h after (‘treatment model’) subcutaneous injection of smac mimetic. Outcome assessment was performed on day 1 (histology) and day 3 (clinical score). **d**–**j**, Clinical assessment (**d**,**g**,**h**), histology (**e**), dermal thickness (**f**; 15 measurements per mouse), lesion size (**i**) and change in body weight (**j**) in the pre-treat (**d**–**g**) and treatment models (**h**–**j**). **e**, Insets show magnification of indicated area. Scale bars, 100 μM. Number of mice per cohort as indicated; each data point represents 1 mouse; 2 independent experiments; representative images are shown. **j**, Weight change data are mean ± s.e.m. Cl-CASP3, cleaved caspase-3; H&E, haematoxylin and eosin; SM, smac mimetic; veh, vehicle. **k**, Schematic of oral JAKi treatment in the humanized mouse model of TEN. **l**–**n**, Ocular reaction (**l**, black arrows), disease occurrence (**m**) and percentage of subepithelial cell death (**n**) in the corresponding cohort following daily culprit-drug administration with or without oral baricitinib (10 mg kg^−1^). Number of mice as indicated. **d**,**f**–**j**, One-tailed unpaired Welch’s *t*-test. **n**, Unpaired two-sided *t*-test. **a**,**c**,**k**, Partially created with Biorender.com.

To investigate the clinical significance of our experimental findings, we developed a novel in vitro model to authentically replicate key aspects of cutaneous drug reactions^42^ (Fig. 5a). In this autologous co-culture model, activated peripheral blood mononuclear cells (PBMCs) efficiently killed keratinocytes within 72 h, and this effect was inhibited in a dose-dependent manner by the pan-JAK inhibitor tofacitinib (Fig. 5b).

**Extended Data Fig. 7:**
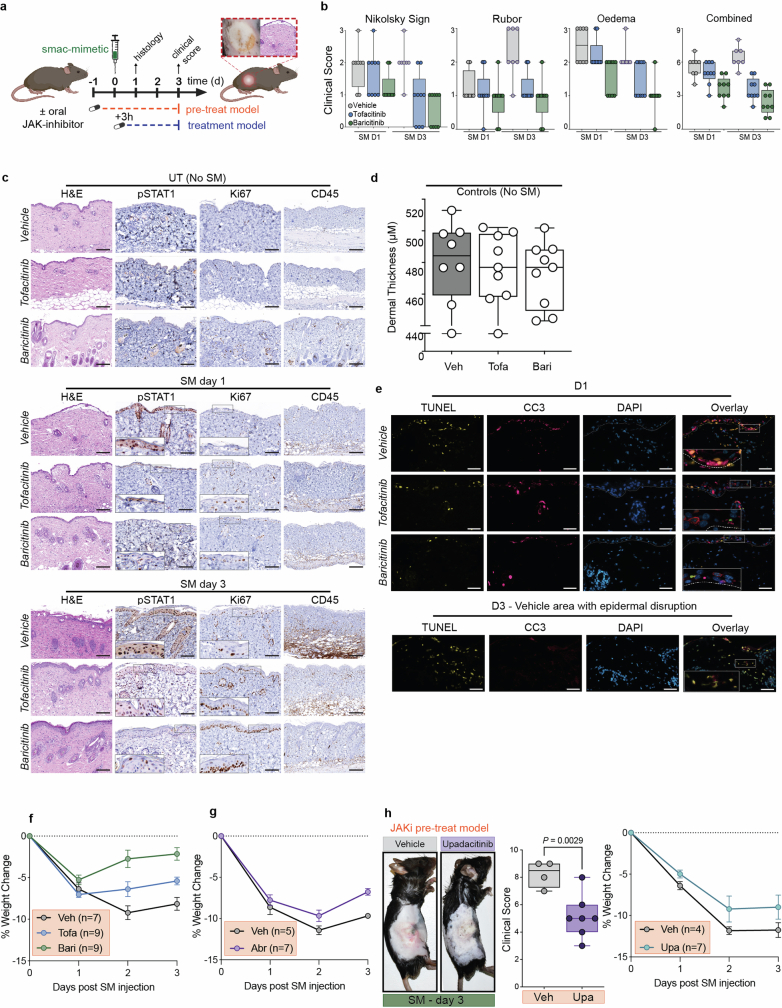
JAK inhibition in smac-mimetic mouse model of TEN. **a**, Schematic experimental protocol for assessing JAK inhibitor efficacy in the smac-mimetic (SM) induced TEN model. Evaluation on day 1 (D1, histology) and day 3 (D3, clinical score). JAK inhibitors (10 mg/kg Baricitinib, 30 mg/kg Tofacitinib, 20 mg/kg Abrocitinib or 10 mg/kg upadacitinib) or vehicle (20% Captisol, 50 μl) were given orally twice daily. Partially created with Biorender.com. **b**, Individual and combined clinical scores for the three assessment criteria (Epidermal disruption, Rubor, Oedema) evaluating clinical severity on D1 and D3 after SM-injection. **c**. Representative histology (H&E, pSTAT1, Ki67 and CD45) of the corresponding cohorts without SM (UT, untreated) and on D1 and D3 post-SM. Insets magnify indicated area of interest. Scale bars = 100 µM. **d**, Dermal thickness of the control cohorts, without SM. Each data point represents 1 mouse, n = 15 measurements/mouse. **e**, Immunofluorescence staining for cell death in the indicated cohorts following SM injection. Dotted lines indicate the dermo-epidermal junction. **b-e**, n = 7 (Vehicle D3), 8 (Vehicle D1) or 9 (tofacitinib and baricitinib) mice examined over 4 independent experiments (2 each D1 & D3 cohorts). **f,g**, Percentage weight change over three days post SM injection in the corresponding treatment groups. **h**, Representative macroscopic image, clinical assessment score and relative weight change following SM injection and pretreatment with upadacitinib (n = 7) or vehicle (n = 4). Weight change data points (**f, g, h**) show mean +/− SEM. **h**, one-tailed Welch t-test. All box plots: median (center), interquartile range (box, 25th-75th percentiles), whiskers (furthest points within 1.5 * interquartile range); Veh, vehicle; Tofa = tofacitinitb; Bari = baricitinib; Abr = abrocitinib.

Encouraged by these results, we next evaluated the efficacy of oral JAKi in established mouse models of TEN^22,43–47^. Subcutaneous injection of a small-molecule antagonist of inhibitor of apoptosis (IAP) proteins (second mitochondria-derived activator of caspases (smac) mimetic) induced TEN-like cutaneous inflammation, epidermal detachment and histological features of TEN (Fig. 5c–f). Oral treatment with the pan-JAK inhibitor tofacitinib or the JAK1 and JAK2 inhibitor baricitinib successfully reduced macroscopic disease severity on days 1 and 3 of treatment (Fig. 5d and Extended Data Fig. 7a,b). Mirroring human TEN, a strong increase in cutaneous phosphorylated STAT1 signal was detectable, which was significantly reduced upon JAKi treatment (Fig. 5e and Extended Data Fig. 7c). Similarly, average dermal thickness was restored to normal levels (Fig. 5f and Extended Data Fig. 7d). Further, JAKi decreased keratinocyte cell death (TUNEL^+^cleaved caspase-3^+^ cells; Fig. 5e and Extended Data Fig. 7e) and reduced immune cell infiltration (CD45^+^ cells; Extended Data Fig. 7c). Of note, epidermal recovery was accelerated in JAKi-treated mice, as demonstrated by the rapid clinical recovery and upregulated expression of the proliferation marker Ki67 in keratinocytes of the basal layer (Extended Data Fig. 7c,f). This argues against detrimental effects of JAKi treatment on wound healing in the context of TEN.

As JAK1 is the primary mediator of interferon signalling, we tested whether inhibition of JAK1 alone would be sufficient to treat the disease. Indeed, treatment with the JAK1 inhibitors abrocitinib or upadacitinib significantly reduced disease severity (Fig. 5g and Extended Data Fig. 7g,h). We also explored a therapeutic model in which JAKi was administered after disease induction (Fig. 5h). This similarly significantly reduced clinical severity, lesion size and time of recovery (Fig. 5h–j).

**Extended Data Fig. 8:**
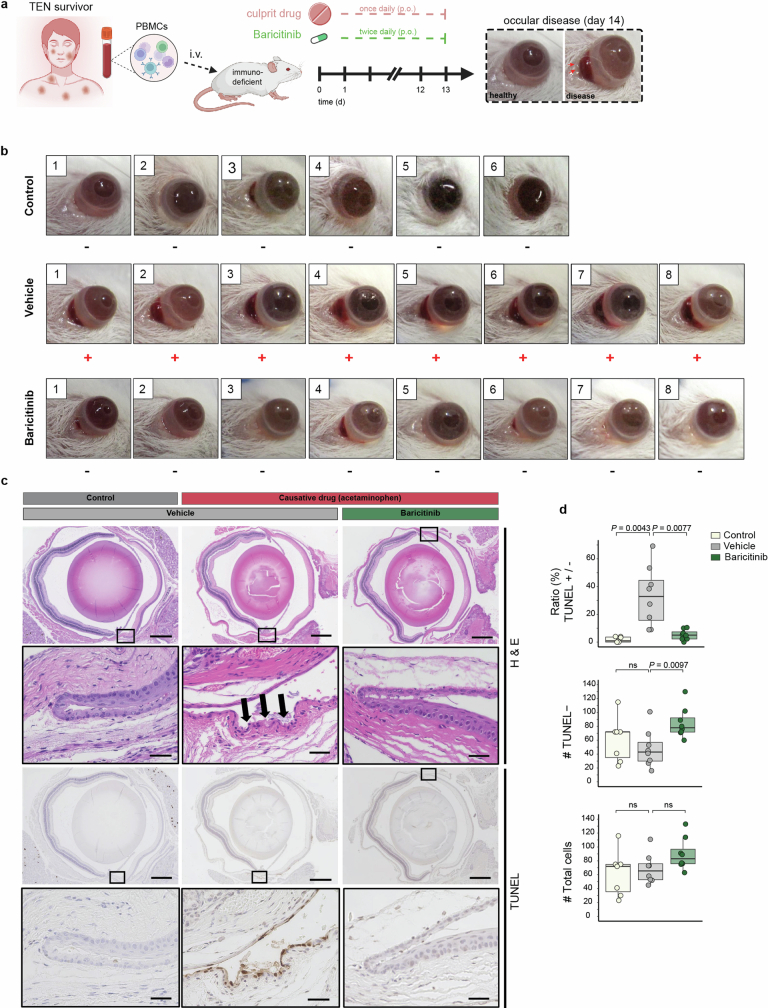
JAK inhibition in humanized mouse model of TEN. **a**, Schematic of oral JAK inhibitor treatment in the humanized mouse model of TEN. Partially created with Biorender.com. **b**, Ocular reaction following daily culprit drug administration ± oral baricitinib (10 mg/kg) with a plus or minus under each picture depending on the occurrence of an immune-reaction. **c**, Representative H&E and TUNEL immunohistochemistry images of the murine eye (scale bars = 500μm) across the cohorts. Insets magnify the conjunctival subepithelial area of interest (scale bars = 50 μm). Arrows indicate epithelial cell death (TUNEL +). **d**, Quantification of TUNEL positive, TUNEL negative, their ratio and total count of subepithelial cells of the corresponding cohort. Each data point is a mouse. Box plots: median (center), interquartile range (box, 25th-75th percentiles), whiskers (furthest points within 1.5 * interquartile range). Unpaired, two-sided t-test. **b-d**, n = 6 control, 8 vehicle and 8 baricitinib treated mice.

We further tested the efficacy of baricitinib in an established humanized mouse model of TEN, in which we injected PBMCs from a TEN survivor intravenously into immunodeficient mice (Fig. 5k). Severe ocular conjunctivitis and cell death of conjunctival epithelium reminiscent of TEN occurred at day 14 following daily oral administration of the causative drug^22^ (Fig. 5l–n and Extended Data Fig. 8a–c). Baricitinib treatment significantly reduced ocular conjunctivitis and cell death of conjunctival epithelium compared with vehicle treatment (Fig. 5l–n and Extended Data Fig. 8a–d). Together, the in vitro and in vivo data clearly demonstrate the efficacy of JAKi in preclinical models of TEN.

## JAKi treatment resolves TEN

**Fig. 6:**
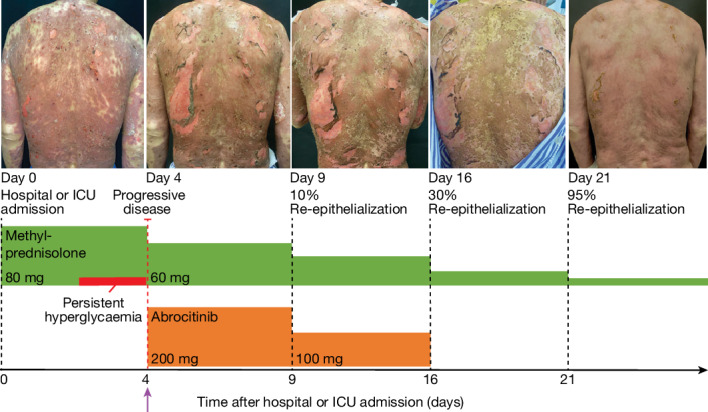
Beneficial effect of JAK/STAT inhibition in patients with TEN. Disease course in a patient with TEN (SCORTEN 4) associated with cancer treatment. Disease progression was observed during high-dose intravenous methylprednisolone treatment and the patient developed persistent hyperglycaemia. JAK1i rescue therapy with abrocitinib was initiated on day 4, resulting in visible cessation of progression within 48 h and initial re-epithelialization within 4 days. Top, photographs of the back of the patient, showing the degree of re-epithelialization at the indicated timepoints after hospital admission. Bottom, Treatment schedule. Arrow marks start of abrocitinib treatment.

**Extended Data Fig. 9:**
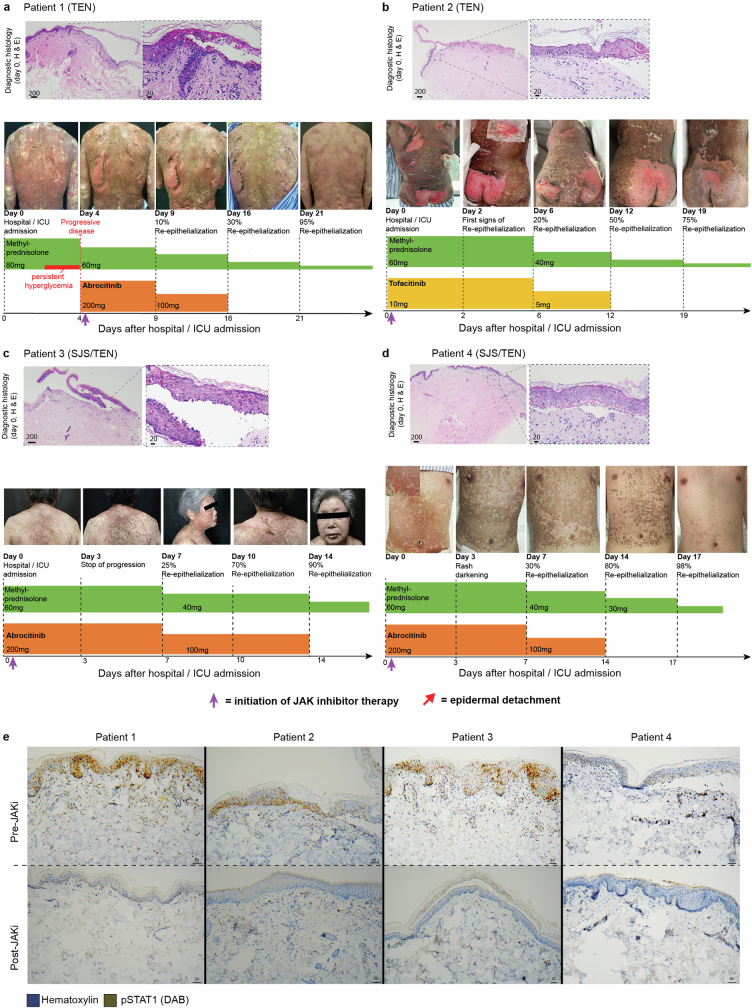
JAK inhibition in 4 patients with TEN and SJS/TEN. **a**, 45-year-old male as described in Fig. 6. **b**, 67-year-old male with hepatocellular carcinoma under checkpoint inhibitor (Tislelizumab) therapy with clinically and histologically confirmed toxic epidermal necrolysis (TBSA 35%, SCORTEN 3, predicted mortality 35.3%). Methylprednisolone (1 mg/kg/day) and tofacitinib (10 mg/day for six days, 5 mg/day for six days) was given. Rapid cessation of disease progression was observed followed by 20% re-epithelialization of detached skin areas six days later. **c**, 72-year-old female with clinically and histologically confirmed Stevens-Johnson-Syndrome / TEN overlap syndrome (TBSA 10%, SCORTEN 2, predicted mortality 12.1%) associated with allopurinol intake. Upon admission she was immediately treated with methylprednisolone (1 mg/kg/day) and abrocitinib (200 mg/day for seven days, 100 mg/day for seven days) resulting in cessation of progression with regression of erythema within three days and 25% re-epithelialization of detached skin areas within seven days. **d**, 18-year-old male with clinically and histologically confirmed Stevens-Johnson-Syndrome/TEN overlap syndrome (TBSA 12%, SCORTEN 1, predicted mortality 3.2%) associated with allopurinol intake. Upon admission, the patient was treated with methylprednisolone (1 mg/kg/day) and abrocitinib (200 mg/day for seven days, 100 mg/day for seven days), resulting in cessation of progression with regression of erythema within three days and 30% re-epithelialization of detached skin areas by day seven. All H&E stained FFPE lesional skin biopsies were taken on day 0 (timepoint of hospital admission). **e**, pSTAT1 staining before and after JAK-inhibitor treatment, per patient. TBSA, total body surface area. Scale bars as indicated [μm].

**Extended Data Fig. 10:**
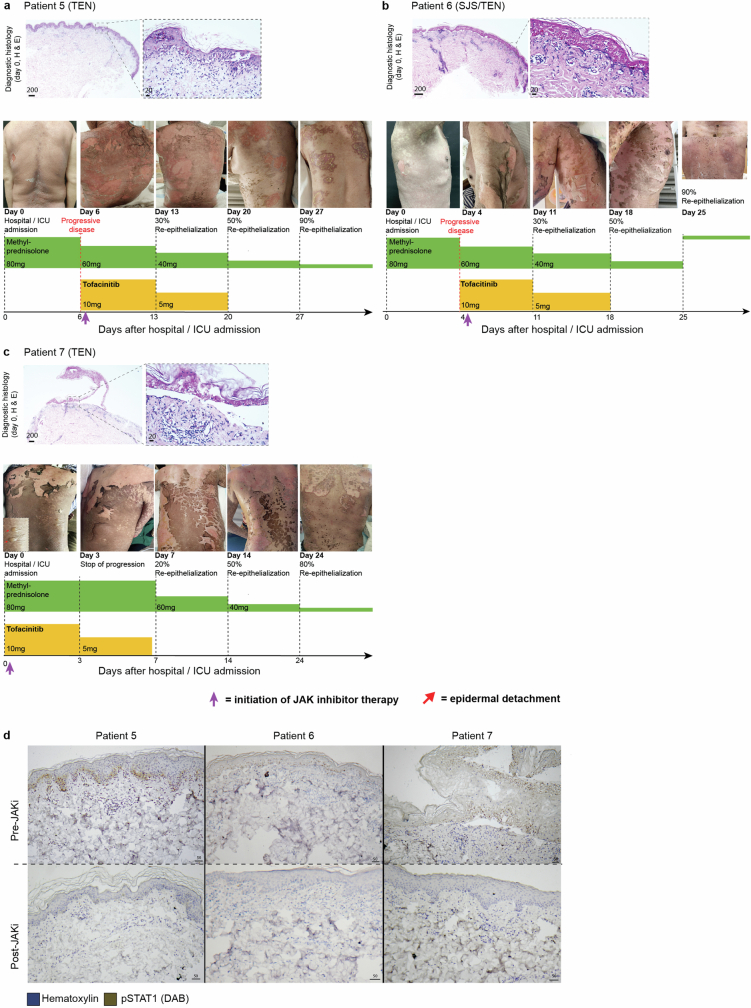
JAK inhibition in 3 patients with TEN and SJS/TEN. **a**, 67-year-old female with clinically and histologically confirmed toxic epidermal necrolysis (TBSA 35%, SCORTEN 2, predicted mortality 12.1%) associated with metronidazole intake, unresponsive to methylprednisolone (1 mg/kg/day) monotherapy was given tofacitinib (10 mg/day for seven days, 5 mg/day for seven days). Rapid cessation of disease progression was observed followed by 30% re-epithelialization of detached skin areas seven days later. **b**, 45-year-old male with clinically and histologically confirmed Stevens-Johnson-Syndrome /TEN overlap syndrome (TBSA 25%, SCORTEN 5, predicted mortality >90%) associated with levofloxacin, cefixime intake, unresponsive to methylprednisolone (1 mg/kg/day) monotherapy was given tofacitinib (10 mg/day for seven days, 5 mg/day for seven days). Rapid cessation of disease progression was observed followed by 30% re-epithelialization of detached skin areas seven days later. **c**, 64-year-old male with laryngeal cancer under checkpoint inhibitor (Zimberelimab) therapy clinically and histologically confirmed toxic epidermal necrolysis (TBSA 30%, SCORTEN 3, predicted mortality 35.3%) associated with carboplatin. Upon admission, he was given methylprednisolone (1 mg/kg/day) and tofacitinib (10 mg/day for seven days, 5 mg/day for seven days). Rapid cessation of disease progression was observed three days later and followed by 20% re-epithelialization of detached skin areas seven days later. All H&E stained FFPE lesional skin biopsies were taken on day 0 (timepoint of hospital admission). **d**, pSTAT1 staining before and after JAK-inhibitor treatment, per patient. TBSA, total body surface area. Scale bars as indicated [μm].

On the basis of our compelling preclinical data and the urgent clinical need in this devastating disease, we treated seven patients with TEN or SJS–TEN overlap with JAKi off-label. One such individual was a 59-year-old man who was receiving carboplatin, etoposide and the PD1 antagonist serplulimab for small cell lung cancer (Fig. 6). After the third cycle of cancer treatment, he developed TEN with epidermal detachment affecting 35% of his body surface and a severity-of-illness score (SCORTEN^48^) of 4, indicating a predicted mortality rate of 58.3%. Epidermal detachment had progressed despite high-dose intravenous methylprednisolone, prompting us to initiate JAK1 inhibitor rescue therapy with abrocitinib, which was recently approved for treatment of atopic dermatitis^49^. The disease stopped progressing within 2 days and re-epithelialization was visible within four days, reaching near-completeness after 16 days (95%), after which the patient was discharged following full recovery from TEN. All seven patients treated with JAKi survived at day 30 with no side effects (Extended Data Figs. 9a–d and 10a–c). Notably, cutaneous levels of phosphorylated STAT1 were markedly reduced in all seven patients following JAKi treatment (Extended Data Figs. 9e and 10d). These clinical data show the potential of JAK1 or pan-JAK inhibition for the treatment of TEN.

## Discussion

Here we used DVP to characterize the spatial proteome of keratinocytes and immune cells in FFPE tissue sections from patients with drug-induced skin reactions, quantifying up to around 5,000 proteins per cell type. These proteomic analyses across all major types of cutaneous drug reactions with cell-type resolution provided insights into the molecular mechanism of each CADR that we leveraged for therapeutic interventions.

Proteomics analysis revealed an in-depth view of the biological molecules involved in CADRs. For instance, the histone methyltransferase EZH2 was detected exclusively in skin-infiltrating immune cells in DRESS. EZH2 has a key role in epigenetic gene regulation by modifying histone H3 at lysine 27 (H3K27me3). In addition, E2F pathway enrichment detected in our proteomic dataset of immune cells in DRESS correlates with overexpression of EZH2, which functions as a downstream effector in this signalling cascade^50^. These findings suggest a role of EZH2, and by extension epigenetic modifications, in the pathogenesis of DRESS that would be of interest for future studies.

The key finding of our study is the marked upregulation of the JAK/STAT pathway in TEN, which is not observed in less severe CADRs. Our results pinpoint this signalling pathway as the main driver of cutaneous inflammation, keratinocyte cytotoxicity and epidermal detachment, with macrophages showing the greatest inflammatory response among tissue immune cells. Whereas activation of STAT1 and STAT2 specifically indicates interferon signalling, STAT3 can be activated by cytokines other than interferons. Our spatial analysis revealed pronounced inflammation in keratinocytes, both at the site of epidermal detachment and immediately adjacent to it. This observation aligns closely with clinical findings showing that light pressure near an existing blister induces further blistering (‘indirect Nikolsky sign’). It also suggests an active role for keratinocytes in contributing to the skin-directed cytotoxic immune response. Upregulation of JAK/STAT signalling in both immune cells and keratinocytes may explain the extensive tissue damage in TEN through a potentially detrimental self-amplifying inflammatory cascade^51^. Further study of macrophages in this context could clarify the role of the innate immune system in TEN. Finally, we comprehensively investigated the effect of JAKi in vitro in a novel cell culture model and in vivo in two independent mouse models, all of which demonstrated consistent therapeutic benefits.

Having identified the JAK/STAT pathway as an actionable therapeutic target in TEN using spatial proteomics, and given our extensive preclinical evidence, we treated seven patients with TEN or SJS–TEN overlap syndrome, including three who were previously unresponsive to high-dose systemic corticosteroids. All seven responded well to JAKi and were discharged in good health after treatment. These data pave the way for a clinical trial of early JAKi therapy in TEN with the potential to change the outcome of this lethal adverse reaction to common drugs.

Owing to the immediate risk of mortality associated with TEN and the short duration of specific therapy required, the rapid onset of action and benefits of short-term JAKi therapy would be substantial, if they are confirmed in larger clinical trials. Given the strong association of TEN with immune checkpoint inhibitor therapy^52^ (odds ratio = 4.33), JAKi may also prove useful in such cases by improving outcome and enabling rapid reintroduction of oncological treatment^53,54^.

Our study is a successful example of directly linking emerging cell-type-resolved spatial omics technologies, especially spatial proteomics, with a new treatment modality that is of benefit to patients. We envision that similar approaches could be transformative in a broad set of inflammatory or oncological conditions by helping to identify key druggable targets and select treatments more precisely.

## Methods

### Patient biopsies

Skin tissue biopsies were obtained during routine histopathological diagnostic procedures. In the standard DVP proteomic cohort, patients with TEN had a minimal affected skin area of 30%. In the other cohorts, patients with SJS–TEN overlap were included (minimal affected skin >10%). All patients with DRESS had a RegiSCORE of 6–7 (definite diagnosis). In addition to the typical clinical features, the lymphocyte transformation test or skin tests were positive in all patients with MPR. The number of samples per condition is indicated in the relevant figures and corresponding legends. Each sample represents one individual. Exceptions are the phosphoproteomic cohort where two distinct biopsies from each of the four healthy individuals yielded eight samples, and the mDIA–DVP cohort, where two distinct biopsies from three out of seven patients resulted in a total of ten samples. Histopathological diagnosis was re-validated in all cases by a board-certified dermatopathologist using a fresh H&E-stained tissue section. Baseline characteristics of all cohorts are described in Supplementary Tables 1–5 and were statistically evaluated using ANOVA for numeric variables (age) and a Chi-squared test of independence for categorical variables (sex). Retrospective analysis was performed with informed consent and ethical approval in place (Munich: 22-0342, 22-0343; Zurich: BASEC: Req-2021-00226 and 2017--00494; Fujian: MRCTA, ECFAH of FMU[2023]400). All experiments were performed in accordance with the Declaration of Helsinki.

### Treatment of patients with JAKi

Regular assessment and vigilant surveillance of the vital signs, haematological parameters and coagulation markers was performed throughout treatment. Patients with active infections were excluded. All patients were additionally treated according to current best supportive care guidelines^25^. Oral abrocitinib was administered at a regimen of 200 mg daily for 5–7 days, and tapered to 100 mg daily for 5–7 days. Similarly, oral tofacitinib was administered at a dosage of 10 mg daily for 5–7 days, and then tapered to 5 mg daily for 5–7 days. In cancer patients with high SCORTEN, a reduction in JAKi dosage was initiated after observing a certain degree of re-epithelialization. Treatment was approved by the local ethics committee and the institutional review board of the First Affiliated Hospital of Fujian Medical University (Fujian: MRCTA, ECFAH of FMU[2023]400), and the patient provided written informed consent.

### Mice

#### Smac-mimetic induced TEN mouse model

As previously published^43^, C57BL/6 mice were injected subcutaneously with 100 μl of 1 mg ml^−1^ smac mimetic (CompA) (TetraLogic Pharmaceuticals) or vehicle (12% Captisol). Weight dependent doses of JAKi (10 mg kg^−1^ baricitinib, 30 mg kg^−1^ tofacitinib, 20 mg kg^−1^ abrocitinib or 10 mg kg^−1^ upadacitinib; Selleckchem) or vehicle (20% Captisol; 50 μl; Selleckchem) was administered by oral gavage twice daily starting one day before (pre-treat model) or 3 h after (treatment model) subcutaneous injection of smac mimetic. Mice were euthanized on day one or day three after smac-mimetic injection, the site was photographed, scored, and samples collected for ex-vivo analysis. We used a four-point ordinal scale (0–4) to assess the three clinical associated macroscopic criteria of TEN (Oedema, rubor and epidermal disruption (Nikolksy sign/lesion severity)) at day one or three after smac-mimetic injection. They were assessed and scored as 0 (not present), 1 (mild), 2 (moderate) or 3 (severe) and the scores were combined for an overall clinical score. *P* values were calculated using the unpaired *t*-test with Welch’s correction. The corresponding data can be found in Supplementary Table 13. All pre-treatment experiments were performed using male mice only, and the treatment model was performed using female mice. Mice were maintained at the appropriate biosafety level under constant temperature and humidity conditions with a 12 h light cycle. Animals were allowed food and water ad libitum. Experiments were approved by the local ethics committee (WEHI AEC 2022.009).

#### Humanized mouse model of TEN

Immunocompromised NOD/Shi-scid, *Il2rg*^*null*^(NOG) mice at 6 weeks of age were included. PBMCs (2 × 10^6^) from an individual who had recovered from SJS–TEN 1 year earlier were injected intravenously into the NOG mice, followed by oral administration of the causative drugs (acetaminophen, 1.5 mg per 100 μl) at day one and once daily thereafter. The dosage used in this model was based on mg per kg body weight, converted from the normal adult human dose. Baricitinib (10 mg kg^−1^) or vehicle (20% Captisol; 50 μl) was administered by oral gavage twice daily from day one. Body weight, ocular reactions or cutaneous changes were assessed daily. On day 14, the degree of conjunctivitis was evaluated after ocular dislocation under general anaesthesia and scored (0, no conjunctivitis; 1, mild conjunctivitis; 2, severe conjunctivitis). The ratio of the number of TUNEL-positive cells to the total conjunctival cell count in a 400× magnified field was calculated for all mice. The corresponding data can be found in Supplementary Table 14. The patients received no systemic glucocorticoids at the time of PBMCs collection. Mice were maintained at the appropriate biosafety level under constant temperature and humidity conditions with a 12 h light cycle. Animals were allowed food and water ad libitum. Experiments were approved by the local ethics committee and the institutional review board of Niigata University, and the patient provided written informed consent.

### Membrane slide tissue mounting, immunofluorescence staining, imaging and laser microdissection

Two-micrometre PEN membrane slides (MicroDissect GmbH) were exposed to UV light (254 nm) for one hour and then coated with Vectabond (Vector laboratories; SP-1800-7) according to the manufacturers protocol. Three-micrometre-thick tissue sections of each FFPE block were mounted on the pretreated membrane slides and dried overnight at 37 °C. Immunofluorescence was performed according to our previously optimized protocol for membrane slides^55^. In brief, slides were heated to 56 °C for 20 min and immediately deparaffinized and rehydrated (Xylol 2× 2 min, 100% ethanol 2× 1 min, 90% ethanol 2× 1 min, 75% ethanol 2× 1 min, 30% ethanol 2× 1 min, ddH_2_O 2× 1 min). Slides were then transferred to prewarmed glycerol-supplemented Antigen Retrieval buffer^55^ (DAKO pH9 S2367 + 10% Glycerol) at 88 °C for 20 min, followed by a 20 min cooldown at room temperature. Slides were then washed in water and blocked with 5% BSA/PBS for 30 min, followed by overnight primary antibody incubation at 4 °C (CD45 or cytokeratin) or 90 min at room temperature (CD4, CD8 and CD163; cytokeratin and CD163) in a humid staining chamber. After washing in PBS, secondary antibodies were incubated for 1 h at room temperature. All primary and secondary antibodies used reported in the Supplementary Table 6. After washing in PBS, sections were counterstained with SYTOX Green (1:400, Invitrogen S7020) for 10 min, washed again and coverslipped using Slowfade Diamond Antifade Mountant (Invitrogen, S36963). Sections were imaged on a Zeiss Axioscan at 20× magnification with a tile overlap of 10%. At the corresponding excitation wavelength at 100% laser intensity, except for 50% for the 493 nm channel. Illumination time was adapted to the optimal spectral properties. Up to 5 *z*-stacks at an interval of 1.25 μm were acquired. Multi-scene images were then split into single scenes, *z*-stacks were combined to a single plane by extended depth of focus where applicable (variance method, standard settings) and stitched, using the proprietary Zeiss Zen Imaging software. Images were imported as.czi files into the Biological Image Analysis Software (BIAS) with the packaged import tool^7^. Single-cell segmentation for Figs. 1 and 2 was performed using deep neural network on the basis of pan-cytokeratin for keratinocytes and CD45 for immune cells at 1.0 input spatial scaling, 50% detection confidence and 30% contour confidence. Segmentation of images for Fig. 3 was performed using a custom-trained Cellpose model^56^. Only contours between 30 μm^2^ and 200 μm^2^ were taken into consideration. After removal of duplicates at tile-overlapping regions, a supervised machine learning approach was used to remove false identifications and overlapping cell types. Contours were exported together with three calibration points that were chosen at characteristic tissue positions. Contour outlines were simplified by removing 99% of data points. For keratinocytes, only maximally every second shape was (randomly) chosen in order to prevent membrane instability while cutting multiple adjacent cells. Contour outlines were imported after reference point alignment, and shapes were cut by laser microdissection with the LMD7 (Leica) with a 63× objective in a semi-automated manner at the following settings: power 57, aperture 1–2, speed 23, middle pulse count 1, final pulse −3, head current 46–53%, pulse frequency 2,600, offset 210.

### Sample preparation, LC–MS/MS (TimsTOF) and raw data analysis for standard DVP

For proteomic analysis of CD45^+^ immune cells and pan-CK^+^ keratinocytes, we collected 700 contours for each keratinocyte sample and 1,000 for each immune cell sample. For each participant, biological duplicates were collected when possible, from the same slide at different regions. Dissected contours for each participant and cell type were collected directly into the same underlying low-binding 384-well plate (Eppendorf 0030129547), with immune cells in the top half of the plate and keratinocytes in the bottom half, leaving an empty well between each sample.

#### Sample preparation

Semi-automated sample preparation and digestion was performed in the collection plate using a Bravo pipetting robot (Agilent) as previously described^7^. For this, wells were washed with 28 μl of 100% acetonitrile and dried in a SpeedVac for 20 min at 45 °C. The contours were then resuspended in 4 μl of 60 mM triethylammonium bicarbonate (TEAB, Sigma) in mass spectrometry-grade H_2_O, sealed with two adhesive foils and heated at 95 °C for 60 min in a 384-well thermal cycler (Eppendorf). 1 μl of 60% acetonitrile in 60 mm TEAB (12.5% final acetonitrile concentration) was added, followed by heating at 75 °C for 60 min in a 384-well thermal cycler. Samples were then predigested with 4 ng of trypsin for 4 h followed by overnight digestion with 6 ng LysC in a 384-well thermal cycler at 37 °C. After 18 h the reaction was quenched with 1.5 µl 6% trifluoroacetic acid (1% final concentration). Samples were then manually transferred to single PCR tubes, dried in a SpeedVac for approx. 60 min at 45 °C and stored at −20 °C.

#### LC–MS/MS analysis

Peptides were resuspended in 4.1 μl MS loading buffer (2% acetonitrile (v/v) + 0.1% trifluoroacetic acid (v/v) in mass spectrometry-grade H_2_0) immediately prior to measurement. All samples were stratified into cell type and replicates, and further randomized using the rand function in Excel. An EASY nanoLC 1200 (Thermo Fisher Scientific) was coupled to a timsTOF SCP mass spectrometer (Bruker) via a nanoelectrospray ion source (Captive spray source, Bruker). Peptides were separated on a 50 cm in-house packed HPLC column (75 μm inner diameter, 1.9 μm ReproSil-Pur C18-AQ silica beads (Dr. Maisch)), which was heated to 60 °C by an in-house manufactured oven. A linear gradient of 120 min was ramped at a constant flow rate of 300 nl min^−1^ from 3 to 30% buffer B in 95 min, followed by an increase to 60% for 5 min, washed at 95% buffer B for 10 min and re-equilibration for 10 min at 5% buffer B (buffer A: 0.1% formic acid and 99.9% ddH_2_O; buffer B: 0.1% formic acid, 80% acetonitrile and 19.9% ddH_2_O). The timsTOF SCP mass spectrometer was operated in dia-PASEF mode using the standard 16 dia-PASEF scan acquisition scheme^57^ with 4 IM steps per dia-PASEF scan, covering a *m*/*z* range from 400 to 1,200 and ion mobility of 0.6 to 1.6 V cm^−2^. All other settings were standard as described in ref. ^58^.

#### Raw data analysis with DIA-NN

A library-free search was performed, using a DL predicted spectral library in DIA-NN (v1.8.0)^59^. Uniprot human databases UP000005640_9606 and UP000005640_9606_additional were used. Mass spectrometry raw files from immune cells and keratinocytes were searched separately in DIA-NN, apart from data shown in Extended Data Fig. 1d–f and Supplementary Fig. 1 (ligand–receptor interaction). Methionine oxidation was defined as variable modification and missed cleavages were limited to one. The precursor charge ranged from 2 to 4, precursor mass range was set to 300 to 1,800, and peptide length from 7 to 35. Mass and MS1 accuracy were set to 15, based on prior estimation. Isotopologues, and MBR were enabled and neural network classifier was set to single-pass mod. The ‘--relaxed-prot-inf’ function was activated using the command line for more conservative protein grouping. Proteins were inferred from FASTA. Library generation was set as ‘Smart profiling’, ‘RT-dependent’ as cross-run normalization an ‘Robust LC (high precision)’ as quantification strategy.

### Sample preparation, LC–MS/MS (Orbitrap Astral) and raw data analysis for mDIA–DVP

For proteomic analysis of different immune cell subsets (CD163^+^ macrophages, CD8^+^ and CD4^+^ T cells) and spatially resolved keratinocytes, we collected ~35 contours for each keratinocyte sample and ~100 for each immune cell sample (equivalent to 5,000 µm^2^) into an underlying 384-well plate and further processed.

#### Sample preparation

Dimethyl labelling of reference channel (∆0) and samples (laser microdissected macrophages, CD8^+^ T cells, CD4^+^ T cells or keratinocytes; ∆8) was performed as previously described^33^. For the reference channel, an equal amount (measured prior using the tryptophan assay) of digested bulk TEN tissue (5 µm FFPE section), tonsil tissue (5 µm FFPE section) and primary keratinocytes (cell culture) was combined and diluted to 1 ng µl^−1^. Each sample was loaded together with 10 ng of the reference channel on an Evotip Pure (Evosep).

#### LC–MS/MS analysis

Peptides were separated by the Evosep One liquid chromatography system and the standardized whisper 40SPD connected to the Orbitrap Astral (Thermo) using the EASY-Spray source (Thermo). Spray voltage was set to 1,900 V. The 15 cm column with an inner diameter of 75 µm containing 1.7 µm C18 beads (Aurora Elite TS, IonOpticks) was heated to 50 °C. All samples on the Orbitrap Astral were acquired in data-independent acquisition (DIA) mode at an MS1 resolution of 240,000 and scan range of 380−980 *m*/*z*. Normalized AGC target was 500%. Isolation windows of 8 Th scanning with a maximum injection time of 16 ms were recorded. Isolated ions were fragmented at an HCD collision energy of 25%. All samples were acquired with field asymmetric ion mobility spectrometry (FAIMS) enabled at a compensation voltage of −40 V. Gas flow was reduced to 2.5 l min^−1^.

#### Raw data analysis

The DIA-NN search was performed using a custom spectral library and postprocessed with Refquant as described previously in detail^33^. The ‘Channel.Q.Value’ cut-off was likewise set to 0.15. The spectral library was created by gas-fractionation of the bulk samples used for the reference channel (10 fractions, 50 ng each, same mass spectrometry method as above). The output of Refquant was then used for further bioinformatic analysis^33^.

### Phosphoproteomic workflow, LC–MS/MS (Orbitrap Astral) and data analysis

Frozen tissue samples stored in RNA Later at −80 °C were transferred with as little RNA Later as possible to single tissueTUBE TT1 (Covaris) and immediately pulverized using a chilled hammer. Five-hundred microlitres of PreOmics Lysis Buffer was then added, resuspended and transferred to a 1.5 ml Eppendorf tube, followed by boiling at 95 °C for 60 min and protein concentration measurement (Tryptophan assay). To remove any potential remnants of RNA Later that could inhibit tryptic digestion, proteins were transferred to a new 1.5 ml Eppendorf tube using Resyn magnetic beads (Hydroxyl modified, 20 µg µl^−1^ stock) at a protein to bead ratio of 1:5 and according to the manufacturers protocol. Samples and beads were resuspended in 200 µl 60 mM TEAB followed by overnight tryptic digestion(LysC and Trypsin at a protein to enzyme ratio of 1:100) at 37 °C and 1,200 rpm. Next day, enzyme activity was quenched using trifluoroacetic acid (1% final concentration) and peptide concentration was again measured. Twenty micrograms from each sample was transferred to a low-bind 96-well Eppendorf plate followed by fully automated phospho enrichment on the Agilent AssayMAP Bravo using High-Capacity Fe(iii)-NTA cartridges and elution in 500 mM ammonium hydrogen phosphate (pH 4). Enriched peptides were then loaded on Evotip Pure (Evosep). Peptides were eluted into the mass spectrometer using the Evosep One liquid chromatography system and the standardized whisper 40SPD. A 15 cm column with an inner diameter of 75 µm containing 1.7 µm C18 beads (IonOpticks), heated at 50 °C and connected to the Orbitrap Astral (Thermo) using the EASY-Spray source (Thermo). Spray voltage was set to 1,900 V. All samples on the Orbitrap Astral were acquired in DIA mode at an MS1 Orbitrap resolution of 120,000 and scan range of 380−1,180 *m*/*z*. Normalized AGC target was 500%. Isolation windows of 3 Th scanning with a maximum injection time of 5 ms were recorded. Isolated ions were fragmented at an HCD collision energy of 25%. All samples were acquired with field asymmetric ion mobility spectrometry (FAIMS) enabled at a compensation voltage of −40 V. Gas flow was reduced to 2.5 l min^−1^. MS raw files were processed by the Spectronaut software version 17 in directDIA mode (Biognosys) against the human FASTA database (21,039 entries, 2019). Peptides between 7–52 with a FDR less than 1% at the peptide and protein levels were taken into account. Variable modification were defined as serine/threonine/tyrosine phosphorylation, N-terminal acetylation and methionine oxidations. Cysteine carbamidomethylation was defined as a fixed modification. Enzyme specificity was set as Trypsin/P with a maximum of two missed cleavages. The resulting report was then exported and analysed in R. Class I phosphosites were defined as phosphosites with a localization probability > 75% or otherwise defined as non-class I. Functional analysis was performed on the imputed dataset. Redundancies in data, caused by multiplicity, were removed by retaining the ones that have higher fold change and higher –log (p.value), as previously described^60^. *t*-test was performed using the base R function, and the resulting *P* values were adjusted for multiple testing (Benjamini–Hochberg, FDR < 0.05). Gene-centric enrichment analysis was performed using the clusterProfiler package, accessing the MsigDB C5 database. Specifically, overrepresentation analysis was performed on proteins of the corresponding phosphosites proteins upregulated by fold change >0.5, while gene set enrichment analysis was performed on all proteins, including the corresponding fold change. The GOplot package was implemented to generate the circosplot^61^. Phosphosite-specific analysis was performed using the ssGSEA2 package accessing the PTMSigDB and with the default settings^62^.

### Preparation and subsequent transcriptomic analysis of FFPE tissue sections

Ten-micrometre FFPE human skin sections were cut from each sample and deparaffinized using ROTICLEAR (Carl Roth). RNA was isolated using RecoverAll Total NucleicAcid Isolation Kit (AM1975, Thermo Fisher Scientific) and concentrated using the Savant SpeedVac DNA 130 Integrated Vacuum Concentrator (Thermo Fisher Scientific). Concentration was measured using Nanodrop One/Onec (Thermo Fisher Scientific). One-hundred and fifty micrograms of RNA from each sample was hybridized overnight with unique probe pairs for 624 genes, including 594 genes from the nCounter Immunology Panel, 15 internal reference genes, and 30 user defined genes using the Panel Plus option (NanoString Technologies). Data were collected using the nCounter SPRINT Profiler (NanoString Technologies). Normalization was performed using nanostringr v0.4.2.

### Bioinformatics data analysis

Bioinformatics data analysis was carried out using the R statistical computing environment version 4.0.2. For subsequent statistical analysis, protein or gene intensities were log_2_-transformed and average intensities of the biological replicates were computed for each participant and cell type where applicable. Box plots show the median (centre line) with interquartile range of 25% to 75%, whiskers extend to furthest data points within 1.5× the interquartile range from the box boundaries. For the ANOVA, the R stats package was used; variables were zero-centred and scaled prior to analysis. Subsequent multiple testing correction (adj. *P*) was conducted using the Benjamini–Hochberg method with a FDR cut-off of 5%; post hoc Tukey’s HSDs were calculated at a confidence level of 0.95. For differential expression, the non-paired t-test was performed using the rstatix package with standard settings, followed by multiple comparison correction (Benjamini–Hochberg method, FDR 5%) from the stats package. Overrepresentation analyses (ORA) was performed with WebGestalt 2019 (ref. ^63^) and GSEA with the clusterProfiler package (MsigDB H, C2 or C5 database) within the R environment. The heat maps were generated using the pheatmap package, with the data being zero-centred and scaled before display. Only proteins that exhibited an identification rate of 70% or higher in at least one cohort for each cell type independently were included for functional analysis of the proteomic data. Imputation was performed per individual based on a normal distribution (width of 0.3, downshift of 1.8) for principal component analyses, functional analysis of the phosphoproteomic dataset and the mDIA–DVP data. All other statistical analyses were performed on non-imputed data. For the comparison between TEN/DRESS/MPR and healthy for selected proteins in both cell types (shown in Fig. 4b and Extended Data Fig. 3c), a non-paired *t*-test was performed and the log_2_ fold change value of TEN over healthy was visualized in the figure using a consistent range and colour-coding for both cell types. The fill colour of the (semi-circles) was manually adjusted using the ‘eyedropper’ tool in Adobe illustrator.

### Cell–cell interaction analysis

Cell–cell interaction analysis was performed in Python. CK and CD45 files were processed together and loaded via Pandas. Data was filtered to have fewer than 30% missing values on protein level. Missing data points where imputed using scikit-learn’s IterativeImputer with a RandomForestRegressor. Significant genes were identified by performing one-way-anove using Scipy’s stats package and a *P* value cut-off of 0.01. Significant genes were identified by performing a one-way ANOVA using the stats package from SciPy, applying a *P* value cut-off of 0.01. Results were further adjusted by applying a pairwise Tukey test from the Pingouin package, requiring a minimum of five significant comparisons at a Tukey cut-off of 0.05. Protein expression data were averaged by condition. Potential interactions were retrieved from OmniPath (version 0.16.4) and matched by gene name. Interaction plots were generated using CircosPlots. For each potential interaction, the intensity difference was calculated in each direction by subtracting the target intensity from the source intensity. To identify disease-specific interactions, the difference in healthy conditions was subtracted from the difference in disease conditions. The plots were created using the D3Blocks package, and the resulting HTML files were automatically converted to figures using Selenium, CairoSVG, and pdf2image. Only interactions with a delta greater than zero were displayed in the plots. Bubble plots were created using Matplotlib. For each interaction, a matrix was constructed to compare all differences across disease conditions and cell types and plotted as scatterplot. Colour indicates the difference according to a colormap and bubble size reflects the magnitude. Bubble plots for the selected protein interactions across different cell types and conditions are provided in the Supplementary Fig. 1.

### Generation of hair follicle-derived keratinocytes for co-culture assay

Hair follicles were placed on thin-Matrigel (Corning, 354234) coated T25 flasks and covered with 10 μl of 1.8 mg ml^−1^ Matrigel to prevent hair follicle detachment. Following 1 h incubation at 37 °C/5% CO_2_, 1 ml of conditioned mouse embryonic fibroblast medium, from irradiated J2-3T3 cells, supplemented with 10 μM Y-27632 (CST 13624) and 10 ng ml^−1^ FGF2 (Cell Guidance GFH146-50), was carefully added. Medium was changed every 48 h. When outgrowth of keratinocytes became visible, medium was aspirated and replaced with 2 ml Keratinocyte Expansion Medium DermaCult (Stemcell 100-0500, with supplement), and changed every 48 h. When confluent, keratinocytes were detached using TrypLE Express Enzyme (Gibco 12604039) and defined trypsin inhibitor (Gibco R007100) and replated on collagen I (Corning, 354236) coated plates for further expansion.

### Isolation of PBMCs for co-culture assay

PBMCs were isolated using Ficol according to standard protocol. Cells were frozen in liquid nitrogen until further usage. Keratinocyte expansion and PBMC isolation were approved by the local ethics committee with written informed consent (Munich: 23-0544).

### Live-cell imaging co-culture assay

Human PBMCs (2 × 10^5^ per well per 200 μl) were stimulated in U-shape 96-well plates in the presence or absence of CD3/28 (Gibco Dynabeads, 11161D) at a ratio of 1:1, in X-VIVO 15 (Lonza, 881026) for 5 days at 37 °C/5% CO_2_. On day 4 of PBMC stimulation, hair follicle-derived keratinocytes from the same person were plated on collagen I-coated 96-well plates at a density of 8 × 10^3^ per well. On day 5 of PBMC stimulation, keratinocytes were fluorescently labelled (Celltracker Red; Invitrogen C34552) according to the manufacturer’s recommendation. Activated or non-activated (‘resting’) PBMCs were then added to the keratinocytes at a ratio of 1:1, after magnetic removal of CD3/28 beads where necessary. Tofacitinib (CST, 14703) or control (DMSO) was added in the indicated concentration as well as Celltox Green (Promega, G8731, 1:8,000 final dilution) to reach a final volume of 200 μl per well. Every 2 h images were acquired using a live-cell analysis system (Incucyte Sx3, Sartorius) for 72 h at 37 °C/5% CO_2_. Quantification of dead keratinocytes (Celltox^+^Celltracker^+^/total Celltracker^+^) was performed using the proprietary analysis software (Incucyte, Sartorius).

### Immunostaining on regular glass slides

Three-micrometre FFPE tissue sections were mounted on regular Superfrost-Plus glass slides and exposed to high temperatures for improved tissue adherence. Subsequently, sections were deparaffinized and subjected to heat-induced antigen retrieval and blocking solution. The primary antibody was applied to the tissue section for one hour at room temperature or overnight at 4 °C (pSTAT1 and cleaved caspase-3). For immunofluorescence staining, a species-specific secondary antibody coupled to a fluorophore was added for an additional hour, followed by a nuclear counterstain. Alternatively, immunohistochemical staining was conducted using horseradish peroxidase (HRP) coupled secondary antibodies, or signal amplified using biotinylated secondary antibodies followed by labelling with and Avidin–Biotin Conjugate (ABC) HRP Kit. HRP-labelled samples were then colour-developed with 3,3′-diaminobenzidine (DAB) and haematoxylin counterstain. TUNEL staining was performed according to the manufacturer’s instruction prior to application of the anti-cleaved caspase-3 primary antibody for co-staining. H&E staining was performed according to standard protocol. Images were acquired on a Zeiss Axioscan 7 or a DP72 microscope and cellSens imaging software (Olympus), or entire slides were scanned with a 3D Histech Virtual Slide Microscope (Olympus), viewed using CaseCenter (3D Histech) and image captured on a MacBook Pro 13-inch retina display (Ki67, CD45 and H&E). Reagents used are reported in Supplementary Table 6.

### Quantification of STAT1 expression in immunofluorescence images

Three-micrometre-thick FFPE tissue sections of all participants from the proteomic cohort were subjected to multiplex immunofluorescence staining for STAT1, pan-cytokeratin and Hoechst. All stainings were then uniformly acquired as.czi files using the Zeiss Axioscan 7. Single-cell segmentation was performed within QuPath (v0.4.1) using the standard nuclear segmentation algorithm. After segmentation, all available features were extracted, including staining intensities for all channels and nuclei. Segmentation and feature extraction was automated with QuPath using the script editor, to ensure equality. In R (v4.0.2) 5000 nuclei and their corresponding measurements were then randomly chosen per individual and merged into a single file, excluding samples with fewer than 5,000 cells (*n* = 1). To account for staining variation between slides, mean STAT1 fluorescence intensity was divided by its own mean Hoechst intensity on a per cell basis. Statistical significance between cohorts was determined by a unpaired two-sided *t*-test on mean normalized STAT1 values. Data are visualized using ggplot2.

### Reporting summary

Further information on research design is available in the Nature Portfolio Reporting Summary linked to this article.

## Online content

Any methods, additional references, Nature Portfolio reporting summaries, source data, extended data, supplementary information, acknowledgements, peer review information; details of author contributions and competing interests; and statements of data and code availability are available at 10.1038/s41586-024-08061-0.

## Supplementary information

Supplementary Figure 1 Ligand–receptor interaction matrix. Bubble plots for the selected protein interactions across different cell types and conditions. Each grid represents a specific interaction between two proteins, segmented by disease state and cell type. Color represents directionality, size corresponds to difference in average expression levels.

Reporting Summary

Supplementary Tables Supplementary Tables 1–14

## Data Availability

Mass spectrometry and transcriptomic data of this study have been deposited to the ProteomeXchange Consortium via the PRIDE partner repository with the dataset identifier PXD044477. Data tables of all proteomic and transcriptomic measurements performed throughout this manuscript are provided in Supplementary Tables 7–12. Clinical scores, average dermal thickness, lesion size and percentage weight change measurements performed in the smac-mimetic mouse model are provided in Supplementary Table 13. Quantification of subepithelial cell death in the humanized mouse model is provided in Supplementary Table 14. Immunofluorescence images of skin sections from DRESS, TEN, MPR and healthy cohorts stained for CD45 and pan-cytokeratin have been uploaded to BioImage Archive (https://www.ebi.ac.uk/bioimage-archive/; accession number S-BSST1430).

## References

[1] Harris, V., Jackson, C. & Cooper, A. Review of toxic epidermal necrolysis. *Int. J. Mol. Sci.* **17**, 2135 (2016). 27999358 10.3390/ijms17122135 PMC5187935

[2] Sekula, P. et al. Comprehensive survival analysis of a cohort of patients with Stevens–Johnson syndrome and toxic epidermal necrolysis. *J. Invest. Dermatol.* **133**, 1197–1204 (2013). 23389396 10.1038/jid.2012.510

[3] Lazarou, J., Pomeranz, B. H. & Corey, P. N. Incidence of adverse drug reactions in hospitalized patients: a meta-analysis of prospective studies. *JAMA* **279**, 1200–1205 (1998). 9555760 10.1001/jama.279.15.1200

[4] Downey, A., Jackson, C., Harun, N. & Cooper, A. Toxic epidermal necrolysis: review of pathogenesis and management. *J. Am. Acad. Dermatol.* **66**, 995–1003 (2012). 22169256 10.1016/j.jaad.2011.09.029

[5] Hoetzenecker, W. et al. Toxic epidermal necrolysis. *F1000Research* **5**, 951 (2016). 10.12688/f1000research.7574.1 PMC4879934 27239294

[6] Chang, W. C. et al. SJS/TEN 2019: From science to translation. *J. Dermatol. Sci.* **98**, 2–12 (2020). 32192826 10.1016/j.jdermsci.2020.02.003 PMC7261636

[7] Mund, A. et al. Deep Visual Proteomics defines single-cell identity and heterogeneity. *Nat. Biotechnol.* **40**, 1231–1240 (2022). 35590073 10.1038/s41587-022-01302-5 PMC9371970

[8] Rosenberger, F. A. et al. Spatial single-cell mass spectrometry defines zonation of the hepatocyte proteome. *Nat. Methods* **20**, 1530–1536(2023). 10.1038/s41592-023-02007-6 PMC10555842 37783884

[9] Del Pozzo-Magana, B. R. & Liy-Wong, C. Drugs and the skin: a concise review of cutaneous adverse drug reactions. *Br. J. Clin. Pharmacol.* **90**, 1838–1855 (2024). 35974692 10.1111/bcp.15490

[10] Hung, S. I. et al. HLA-B*5801 allele as a genetic marker for severe cutaneous adverse reactions caused by allopurinol. *Proc. Natl Acad. Sci. USA* **102**, 4134–4139 (2005). 15743917 10.1073/pnas.0409500102 PMC554812

[11] Chung, W. H., Hung, S. I. & Chen, Y. T. Human leukocyte antigens and drug hypersensitivity. *Curr. Opin. Allergy Clin. Immunol.* **7**, 317–323 (2007). 17620823 10.1097/ACI.0b013e3282370c5f

[12] Chung, W. H. et al. Medical genetics: a marker for Stevens–Johnson syndrome. *Nature* **428**, 486 (2004). 15057820 10.1038/428486a

[13] Ko, T. M. et al. Shared and restricted T-cell receptor use is crucial for carbamazepine-induced Stevens–Johnson syndrome. *J. Allergy Clin. Immunol.* **128**, 1266–1276.e1211 (2011). 21924464 10.1016/j.jaci.2011.08.013

[14] Nassif, A. et al. Toxic epidermal necrolysis: effector cells are drug-specific cytotoxic T cells. *J. Allergy Clin. Immunol.* **114**, 1209–1215 (2004). 15536433 10.1016/j.jaci.2004.07.047

[15] Le Cleach, L. et al. Blister fluid T lymphocytes during toxic epidermal necrolysis are functional cytotoxic cells which express human natural killer (NK) inhibitory receptors. *Clin. Exp. Immunol.* **119**, 225–230 (2000). 10606987 10.1046/j.1365-2249.2000.01119.x PMC1905549

[16] Friedmann, P. S., Strickland, I., Pirmohamed, M. & Park, B. K. Investigation of mechanisms in toxic epidermal necrolysis induced by carbamazepine. *Arch. Dermatol.* **130**, 598–604 (1994). 8179341

[17] Villada, G., Roujeau, J. C., Clerici, T., Bourgault, I. & Revuz, J. Immunopathology of toxic epidermal necrolysis. Keratinocytes, HLA-DR expression, Langerhans cells, and mononuclear cells: an immunopathologic study of five cases. *Arch. Dermatol.* **128**, 50–53 (1992). 1739287 10.1001/archderm.128.1.50

[18] Heng, M. C. & Allen, S. G. Efficacy of cyclophosphamide in toxic epidermal necrolysis. Clinical and pathophysiologic aspects. *J. Am. Acad. Dermatol.* **25**, 778–786 (1991). 1802900 10.1016/s0190-9622(08)80969-3

[19] Correia, O., Delgado, L., Ramos, J. P., Resende, C. & Torrinha, J. A. Cutaneous T-cell recruitment in toxic epidermal necrolysis. Further evidence of CD8^+^ lymphocyte involvement. *Arch. Dermatol.* **129**, 466–468 (1993). 8466217

[20] Viard, I. et al. Inhibition of toxic epidermal necrolysis by blockade of CD95 with human intravenous immunoglobulin. *Science* **282**, 490–493 (1998). 9774279 10.1126/science.282.5388.490

[21] Paul, C. et al. Apoptosis as a mechanism of keratinocyte death in toxic epidermal necrolysis. *Br. J. Dermatol.* **134**, 710–714 (1996). 8733377 10.1111/j.1365-2133.1996.tb06976.x

[22] Saito, N. et al. An annexin A1-FPR1 interaction contributes to necroptosis of keratinocytes in severe cutaneous adverse drug reactions. *Sci. Transl. Med.* **6**, 245ra295 (2014). 10.1126/scitranslmed.3008227 25031270

[23] Viard-Leveugle, I. et al. TNF-alpha and IFN-gamma are potential inducers of Fas-mediated keratinocyte apoptosis through activation of inducible nitric oxide synthase in toxic epidermal necrolysis. *J. Invest. Dermatol.* **133**, 489–498 (2013). 22992806 10.1038/jid.2012.330

[24] Chung, W. H. et al. Granulysin is a key mediator for disseminated keratinocyte death in Stevens–Johnson syndrome and toxic epidermal necrolysis. *Nat. Med.* **14**, 1343–1350 (2008). 19029983 10.1038/nm.1884

[25] Bruggen, M. C. et al. Supportive care in the acute phase of Stevens–Johnson syndrome and toxic epidermal necrolysis: an international, multidisciplinary Delphi-based consensus. *Br. J. Dermatol.* **185**, 616–626 (2021). 33657677 10.1111/bjd.19893

[26] Vandereyken, K., Sifrim, A., Thienpont, B. & Voet, T. Methods and applications for single-cell and spatial multi-omics. *Nat. Rev. Genet.* **24**, 494–515 (2023). 36864178 10.1038/s41576-023-00580-2 PMC9979144

[27] Mund, A., Brunner, A. D. & Mann, M. Unbiased spatial proteomics with single-cell resolution in tissues. *Mol. Cell* **82**, 2335–2349 (2022). 35714588 10.1016/j.molcel.2022.05.022

[28] Fleckner, J., Martensen, P. M., Tolstrup, A. B., Kjeldgaard, N. O. & Justesen, J. Differential regulation of the human, interferon inducible tryptophanyl-tRNA synthetase by various cytokines in cell lines. *Cytokine* **7**, 70–77 (1995). 7749068 10.1006/cyto.1995.1009

[29] Lerner, L. H., Qureshi, A. A., Reddy, B. V. & Lerner, E. A. Nitric oxide synthase in toxic epidermal necrolysis and Stevens–Johnson syndrome. *J. Invest. Dermatol.* **114**, 196–199 (2000). 10620138 10.1046/j.1523-1747.2000.00816.x

[30] Quinn, A. M. et al. Uncovering histologic criteria with prognostic significance in toxic epidermal necrolysis. *Arch. Dermatol.* **141**, 683–687 (2005). 15967913 10.1001/archderm.141.6.683

[31] Kim, D. et al. Targeted therapy guided by single-cell transcriptomic analysis in drug-induced hypersensitivity syndrome: a case report. *Nat. Med.* **26**, 236–243 (2020). 31959990 10.1038/s41591-019-0733-7 PMC7105105

[32] Chaudhuri, B., Xu, H., Todorov, I., Dutta, A. & Yates, J. L. Human DNA replication initiation factors, ORC and MCM, associate with oriP of Epstein–Barr virus. *Proc. Natl Acad. Sci. USA* **98**, 10085–10089 (2001). 11517328 10.1073/pnas.181347998 PMC56919

[33] Thielert, M. et al. Robust dimethyl-based multiplex-DIA doubles single-cell proteome depth via a reference channel. *Mol. Syst. Biol.* **19**, e11503 (2023). 37602975 10.15252/msb.202211503 PMC10495816

[34] Guzman, U. H. et al. Ultra-fast label-free quantification and comprehensive proteome coverage with narrow-window data-independent acquisition. *Nat. Biotechnol.*10.1038/s41587-023-02099-7 (2024). 10.1038/s41587-023-02099-7 PMC11631760 38302753

[35] Linkermann, A., Stockwell, B. R., Krautwald, S. & Anders, H. J. Regulated cell death and inflammation: an auto-amplification loop causes organ failure. *Nat. Rev. Immunol.* **14**, 759–767 (2014). 25324125 10.1038/nri3743

[36] Levy, D. E., Kessler, D. S., Pine, R., Reich, N. & Darnell, J. E. Jr. Interferon-induced nuclear factors that bind a shared promoter element correlate with positive and negative transcriptional control. *Genes Dev.* **2**, 383–393 (1988). 3371658 10.1101/gad.2.4.383

[37] Tretina, K., Park, E. S., Maminska, A. & MacMicking, J. D. Interferon-induced guanylate-binding proteins: Guardians of host defense in health and disease. *J. Exp. Med.* **216**, 482–500 (2019). 30755454 10.1084/jem.20182031 PMC6400534

[38] Sung, Y., Yoon, I., Han, J. M. & Kim, S. Functional and pathologic association of aminoacyl-tRNA synthetases with cancer. *Exp. Mol. Med.* **54**, 553–566 (2022). 35501376 10.1038/s12276-022-00765-5 PMC9166799

[39] Lewis, C. E., McCarthy, S. P., Lorenzen, J. & McGee, J. O. Differential effects of LPS, IFN-gamma and TNF alpha on the secretion of lysozyme by individual human mononuclear phagocytes: relationship to cell maturity. *Immunology* **69**, 402–408 (1990). 2107146 PMC1385959

[40] Liao, W. et al. A novel anti-apoptotic role for apolipoprotein L2 in IFN-gamma-induced cytotoxicity in human bronchial epithelial cells. *J. Cell. Physiol.* **226**, 397–406 (2011). 20665705 10.1002/jcp.22345

[41] Wang, F. et al. Diverse expression of TNF-alpha and CCL27 in serum and blister of Stevens–Johnson syndrome/toxic epidermal necrolysis. *Clin. Transl. Allergy* **8**, 12 (2018). 29713456 10.1186/s13601-018-0199-6 PMC5909236

[42] Wustner, L. S. Generating iPSCs with a high-efficient, non-invasive method-an improved way to cultivate keratinocytes from plucked hair for reprogramming. *Cells* **11**, 1955 (2022). 35741085 10.3390/cells11121955 PMC9222083

[43] Anderton, H., Rickard, J. A., Varigos, G. A., Lalaoui, N. & Silke, J. Inhibitor of apoptosis proteins (IAPs) limit RIPK1-mediated skin inflammation. *J. Invest. Dermatol.* **137**, 2371–2379 (2017). 28647349 10.1016/j.jid.2017.05.031

[44] Saito, N. et al. Stevens–Johnson syndrome/toxic epidermal necrolysis mouse model generated by using PBMCs and the skin of patients. *J. Allergy Clin. Immunol.* **131**, 434–441 e431-439 (2013). 23111236 10.1016/j.jaci.2012.09.014

[45] Varfolomeev, E. et al. IAP antagonists induce autoubiquitination of c-IAPs, NF-kappaB activation, and TNFalpha-dependent apoptosis. *Cell* **131**, 669–681 (2007). 18022362 10.1016/j.cell.2007.10.030

[46] Vasilikos, L., Spilgies, L. M., Knop, J. & Wong, W. W. Regulating the balance between necroptosis, apoptosis and inflammation by inhibitors of apoptosis proteins. *Immunol. Cell Biol.* **95**, 160–165 (2017). 27904150 10.1038/icb.2016.118

[47] Chung, W. H. & Hung, S. I. Recent advances in the genetics and immunology of Stevens–Johnson syndrome and toxic epidermal necrosis. *J. Dermatol. Sci.* **66**, 190–196 (2012). 22541332 10.1016/j.jdermsci.2012.04.002

[48] Bastuji-Garin, S. et al. SCORTEN: a severity-of-illness score for toxic epidermal necrolysis. *J. Invest. Dermatol.* **115**, 149–153 (2000). 10951229 10.1046/j.1523-1747.2000.00061.x

[49] Bieber, T. et al. Abrocitinib versus placebo or dupilumab for atopic dermatitis. *N. Engl. J. Med.* **384**, 1101–1112 (2021). 33761207 10.1056/NEJMoa2019380

[50] Bracken, A. P. et al. EZH2 is downstream of the pRB–E2F pathway, essential for proliferation and amplified in cancer. *EMBO J.* **22**, 5323–5335 (2003). 14532106 10.1093/emboj/cdg542 PMC213796

[51] Hall, J. C. & Rosen, A. Type I interferons: crucial participants in disease amplification in autoimmunity. *Nat. Rev. Rheumatol.* **6**, 40–49 (2010). 20046205 10.1038/nrrheum.2009.237 PMC3622245

[52] Zhu, J. et al. Stevens–Johnson syndrome/toxic epidermal necrolysis in patients treated with immune checkpoint inhibitors: A safety analysis of clinical trials and FDA pharmacovigilance database. *eClinicalMedicine* **37**, 100951 (2021). 34386743 10.1016/j.eclinm.2021.100951 PMC8343267

[53] Ireland, P. A., Jansson, N., Spencer, S. K. R., Braden, J. & Sebaratnam, D. Short-term cardiovascular complications in dermatology patients receiving JAK-STAT inhibitors: a meta-analysis of randomized clinical trials. *JAMA Dermatol.* **160**, 281–289 (2024). 38294793 10.1001/jamadermatol.2023.5509 PMC10831633

[54] Ytterberg, S. R. et al. Cardiovascular and cancer risk with tofacitinib in rheumatoid arthritis. *N. Engl. J. Med.* **386**, 316–326 (2022). 35081280 10.1056/NEJMoa2109927

[55] Nordmann, T. M. et al. A standardized and reproducible workflow for membrane glass slides in routine histology and spatial proteomics. *Mol. Cell Proteomics* **22**, 100643 (2023). 37683827 10.1016/j.mcpro.2023.100643 PMC10565769

[56] Pachitariu, M. & Stringer, C. Cellpose 2.0: how to train your own model. *Nat. Methods* **19**, 1634–1641 (2022). 36344832 10.1038/s41592-022-01663-4 PMC9718665

[57] Meier, F. et al. diaPASEF: parallel accumulation-serial fragmentation combined with data-independent acquisition. *Nat. Methods* **17**, 1229–1236 (2020). 33257825 10.1038/s41592-020-00998-0

[58] Brunner, A. D. et al. Ultra-high sensitivity mass spectrometry quantifies single-cell proteome changes upon perturbation. *Mol. Syst. Biol.* **18**, e10798 (2022). 35226415 10.15252/msb.202110798 PMC8884154

[59] Demichev, V., Messner, C. B., Vernardis, S. I., Lilley, K. S. & Ralser, M. DIA-NN: neural networks and interference correction enable deep proteome coverage in high throughput. *Nat. Methods* **17**, 41–44 (2020). 31768060 10.1038/s41592-019-0638-x PMC6949130

[60] Al Tarrass, M. et al. Large-scale phosphoproteomics reveals activation of the MAPK/GADD45beta/P38 axis and cell cycle inhibition in response to BMP9 and BMP10 stimulation in endothelial cells. *Cell Commun. Signal.* **22**, 158 (2024). 38439036 10.1186/s12964-024-01486-0 PMC10910747

[61] Walter, W., Sanchez-Cabo, F. & Ricote, M. GOplot: an R package for visually combining expression data with functional analysis. *Bioinformatics* **31**, 2912–2914 (2015). 25964631 10.1093/bioinformatics/btv300

[62] Krug, K. et al. A curated resource for phosphosite-specific signature analysis. *Mol. Cell Proteomics* **18**, 576–593 (2019). 30563849 10.1074/mcp.TIR118.000943 PMC6398202

[63] Liao, Y., Wang, J., Jaehnig, E. J., Shi, Z. & Zhang, B. WebGestalt 2019: gene set analysis toolkit with revamped UIs and APIs. *Nucleic Acids Res.* **47**, W199–W205 (2019). 31114916 10.1093/nar/gkz401 PMC6602449

