# Supplementary figures and images for: Spatial proteomics identifies JAKi as treatment for a lethal skin disease
Source: Nature. 2024 Oct 16;635(8040):1001–9. doi: 10.1038/s41586-024-08061-0 (PMC11602713; doi:10.1038/s41586-024-08061-0)

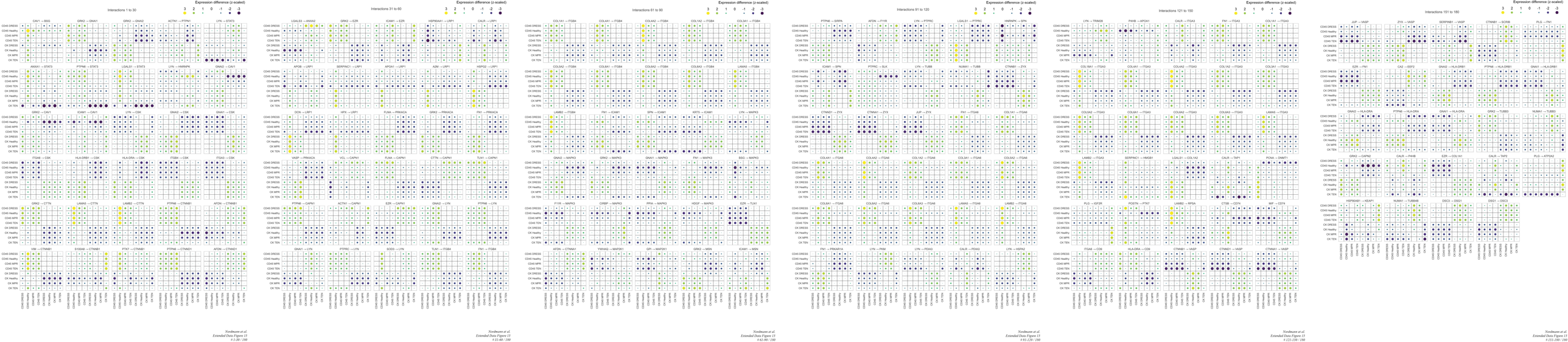

Supplement: Supplementary file 1 — Ligand–receptor interaction matrix. Bubble plots for the selected protein interactions across different cell types and conditions. Each grid represents a specific interaction between two proteins, segmented by disease state and cell type. Color represents directionality, size corresponds to difference in average expression levels. [file 41586_2024_8061_MOESM1_ESM.jpg]
