# Supplementary material for: Spatial proteomics identifies JAKi as treatment for a lethal skin disease
Source: Nature. 2024 Oct 16;635(8040):1001–9. doi: 10.1038/s41586-024-08061-0 (PMC11602713; doi:10.1038/s41586-024-08061-0)
Supplement: Supplementary file 2 — Reporting Summary [file 41586_2024_8061_MOESM2_ESM.pdf]

nature portfolio

Corresponding author(s): Matthias Mann

Last updated by author(s): Aug 17, 2024

Reporting Summary

Nature Portfolio wishes to improve the reproducibility of the work that we publish. This form provides structure for consistency and transparency in reporting. For further information on Nature Portfolio policies, see our Editorial Policies and the Editorial Policy Checklist.

Statistics

For all statistical analyses, confirm that the following items are present in the figure legend, table legend, main text, or Methods section.

n/a	Confirmed	
☐	☒	The exact sample size (*n*) for each experimental group/condition, given as a discrete number and unit of measurement
☐	☒	A statement on whether measurements were taken from distinct samples or whether the same sample was measured repeatedly
☐	☒	The statistical test(s) used AND whether they are one- or two-sided<br>*Only common tests should be described solely by name; describe more complex techniques in the Methods section.*
☐	☒	A description of all covariates tested
☐	☒	A description of any assumptions or corrections, such as tests of normality and adjustment for multiple comparisons
☐	☒	A full description of the statistical parameters including central tendency (e.g. means) or other basic estimates (e.g. regression coefficient) AND variation (e.g. standard deviation) or associated estimates of uncertainty (e.g. confidence intervals)
☐	☒	For null hypothesis testing, the test statistic (e.g. *F*, *t*, *r*) with confidence intervals, effect sizes, degrees of freedom and *P* value noted<br>*Give P values as exact values whenever suitable.*
☒	☐	For Bayesian analysis, information on the choice of priors and Markov chain Monte Carlo settings
☐	☒	For hierarchical and complex designs, identification of the appropriate level for tests and full reporting of outcomes
☐	☒	Estimates of effect sizes (e.g. Cohen's *d*, Pearson's *r*), indicating how they were calculated

Our web collection on statistics for biologists contains articles on many of the points above.

Software and code

Policy information about availability of computer code

Data collection	Zeiss Zen blue (v3.7.97.07000), Incucyte Sx1 (2022B Rev2), Leica LMD (v8.3), Bruker HyStar (v6.0), timsControl (v3.0.20), Evosep One Software (RCNet Driver 2.2.74.0), Orbitrap Astral Tune Application (1.0.100.40), Thermo Scientific Xcalibur (v4.7.69.37), nCounter® SPRINT Profiler (NanoString Tecnhhologies)
Data analysis	DIA-NN (v1.8.0), R(4.0.2), QuPath 0.4.1, Biological Image Analysis Software (BIAS v1.3.0), Python (v3.11), Spectronaut (v17), Cellpose (v2.0), Zeiss Zen Imaging software (ZEN blue v3.7.97.07000), Incucyte analysis software (2022B Rev2), R-packages: ggplot2(3.4.3), pheatmap(v1.0.12), rstatix (v0.7.2), clusterProfiler (v4.6.0), WebGestaltR (v0.4.6), GOplot (v1.0.2), ssGSEA2 (v1.4), nanostringr(v0.4.2); Python-packages: SciPy (v1.11.1), Matplotlib (v3.7.2), Pandas (v2.0.3), Pingouin (v0.5.3), D3Blocks (v1.2.3), OmniPath (v0.16.4)

For manuscripts utilizing custom algorithms or software that are central to the research but not yet described in published literature, software must be made available to editors and reviewers. We strongly encourage code deposition in a community repository (e.g. GitHub). See the Nature Portfolio guidelines for submitting code & software for further information.

Data

Policy information about availability of data

All manuscripts must include a data availability statement. This statement should provide the following information, where applicable:

- Accession codes, unique identifiers, or web links for publicly available datasets
- A description of any restrictions on data availability
- For clinical datasets or third party data, please ensure that the statement adheres to our policy

UniProt human proteome (UP000005640_9606 and UP000005640_9606_additional, https://www.uniprot.org/proteomes/UP000005640), MsigDB C5 and Hallmark database (https://www.gsea-msigdb.org/gsea/msigdb/collections.jsp), PTMSigDB (https://proteomics.broadapps.org/ptmsigdb/), WebGestalt 2019 (http://www.webgestalt.org/), Reactome pathway database (https://reactome.org/), Gene Ontology (GO) database (http://geneontology.org/). Mass spectrometry and transcriptomic data of this study have been deposited to the ProteomeXchange Consortium via the PRIDE partner repository with the dataset identifier PXD044477 (username: reviewer_pxd044477@ebi.ac.uk, password: 6Lhi7MGM). Data tables of all proteomic and transcriptomic measurements performed throughout this manuscript are provided in Supplementary Tables 7 - 12. Clinical scores, average dermal thickness, lesion size and % weight change measurements performed in the smac-mimetic mouse model are provided in Supplementary Table 13. Quantification of subepithelial cell death in the humanized mouse model is provided in Supplementary Table 14.

Research involving human participants, their data, or biological material

Policy information about studies with human participants or human data. See also policy information about sex, gender (identity/presentation), and sexual orientation and race, ethnicity and racism.

Reporting on sex and gender	To study molecular mechanisms of cutaneous drug reactions, we assembled an age and sex matched, retrospective cohort of lesional FFPE skin biopsies for A) proteomics: i) from patients with mild (MPR) or severe (TEN, DRESS) CADRs alongside healthy controls (Fig. 1a and Extended Data Figure 1 / Supplemental Table 1, n = 21), ii) from patients with SJS/TEN and TEN alongside healthy controls (Fig. 3 / Supplemental Table 2, n = 24) and B) transcriptomics: from patients with mild (MPR) or severe (TEN, DRESS) CADRs alongside healthy controls (Fig. 4c-d and Extended Data Fig. 5 / Supplemental Table 3, n = 44). For phosphoproteomics frozen tissue biopsies from patients with SJS/TEN and TEN alongside healthy controls were used (Extended Data Figure 6 / Supplemental Table 4, n = 21).
Reporting on race, ethnicity, or other socially relevant groupings	No socially constructed or socially relevant categorization variables were used in this study.
Population characteristics	Baseline characteristics were statistically evaluated using Analysis of Variance (ANOVA) for numeric variables (age) and a Chi-squared test of independence for categorical variables (sex), a summary of which can be found in the corresponding Extended Data Tables.
Recruitment	Proteomic and transcriptomic analyses were performed on a retrospective cohort. Participants were identified through a review of medical records at the Departments of Dermatology and Allergology of the University Hospital Zurich and Ludwig Maximilian University of Munich. Eligible patients were those who had undergone routine histopathological diagnostic procedures for suspected cutaneous adverse drug reactions (CADRs). Inclusion criteria for the CADR groups were i) TEN patients: a minimal affected skin area of 30% (standard DVP proteomic cohort) or >10% (other cohorts, including SJS/TEN overlap). ii) DRESS patients: a RegiSCORE of 6-7 (definite diagnosis). iii) MPR patients: typical clinical features and a positive lymphocyte transformation test (LTT) or skin test. Healthy control samples were obtained from individuals undergoing routine dermatological procedures unrelated to drug reactions during the same period. We do not assume any self-selection bias in this study due to the comprehensive and systematic approach used in patient identification and sample collection.
Ethics oversight	Ethical permission and informed consent was conducted for all involved patients and samples. Treatment with JAK inhibitors was approved by the local ethics committee and the institutional review board of the First Affiliated Hospital of Fujian Medical University (Fujian: MRCTA, ECFAH of FMU[2023]400), and the patient was provided written informed consent. Skin biopsies were performed in the context of routine clinical workup for retrospective analysis with informed consent and ethical approval in place (Munich: 22-0342, 22-0343; Zurich: BASEC: Req-2021-00226 and 2017--00494; Fujian: MRCTA, ECFAH of FMU[2023]400, all experiments were performed in accordance with the Declaration of Helsinki.

Note that full information on the approval of the study protocol must also be provided in the manuscript.

Field-specific reporting

Please select the one below that is the best fit for your research. If you are not sure, read the appropriate sections before making your selection.

☒ Life sciences ☐ Behavioural & social sciences ☐ Ecological, evolutionary & environmental sciences

For a reference copy of the document with all sections, see nature.com/documents/nr-reporting-summary-flat.pdf

Life sciences study design

All studies must disclose on these points even when the disclosure is negative.

Sample size	Proteomics cohorts n = 45 patients. Phosphoproteomics n = 21 patients. Transcriptomics cohort n = 44 patients. JAK inhibitor treatment n = 7 patients. Mouse cohorts n = 78. For the proteomics, phosphoproteomics, and transcriptomics cohorts, sample size was primarily determined by the availability of rare TEN cases, which naturally limited the cohort size. We first identified available TEN samples that met our inclusion criteria, then added samples from other CADR types and healthy controls to create balanced cohorts, keeping in mind to achieve a number of samples in total that remain processable within the technological aspects. No formal statistical methods were used to predetermine sample sizes. Considering the rarity of the disease and in comparison to existing literature we achieved a high number of analyzed TEN samples that allow the statements made within the manuscript, further supported by the biological follow up experiments. For the JAK inhibitor treatment group, we included all consecutive eligible patients with this rare condition who received the treatment during the study period.
Data exclusions	None.
Replication	We used independent biological samples of different patients per disease (TEN, DRESS, MPR) and across different omic entities. All experiments confirmed the experimental findings. In the phosphoproteomic cohort and mDIA-DVP cohort, 2 different biopsy sites were used in the healthy cohort from a subset of healthy patients, as specified in the methods section.
Randomization	Samples were allocated into the experimental groups based on the type of drug reaction and thus based on the clinical-histological diagnosis. Histopathological diagnosis was re-validated in all cases by a board-certified dermatopathologist using a fresh H&E-stained tissue section. Mass-spectrometry sample injection was randomized using the rand function in Excel.
Blinding	Investigators were not blinded to group allocation during data collection and analysis, as blinding was not feasible due to the nature of the study. Given the distinct clinical and histological features of each type of drug reaction, blinding would not have been practical or meaningful in this context.

Reporting for specific materials, systems and methods

We require information from authors about some types of materials, experimental systems and methods used in many studies. Here, indicate whether each material, system or method listed is relevant to your study. If you are not sure if a list item applies to your research, read the appropriate section before selecting a response.

Materials & experimental systems

n/a	Involved in the study	
☐	☒	Antibodies
☒	☐	Eukaryotic cell lines
☒	☐	Palaeontology and archaeology
☐	☒	Animals and other organisms
☐	☒	Clinical data
☒	☐	Dual use research of concern
☒	☐	Plants

Methods

n/a	Involved in the study	
☒	☐	ChIP-seq
☒	☐	Flow cytometry
☒	☐	MRI-based neuroimaging

Antibodies

Antibodies used	see Supplemental Table 6 for all used antibodies and their usage details.
Validation	Only commercially available antibodies that have been validated for immunohistochemistry as documented on the manufacturer´s website were used. Dilutions used are mentioned in Extended Data Table 8. abcam 228724 / abcam 156769: validated by manufacturer for Western blotting and immunohistochemistry using recombinant human WARS1 protein fragment as immunogen. CST #9167S / CST #5246T / CST #14994 / CST #9661 / CST #98941 / CST #98941 / : validated by manufacturer with Western blotting. DAKO #M0701 / DAKO, #Z0622: validated by manufacturer for immunohistochemistry on formalin-fixed, paraffin-embedded tissues and acetone-fixed frozen sections. LSBio #LS-B16812: validated by manufacturer for immunohistochemistry on formalin-fixed, paraffin-embedded tissues. Thermo Fisher #MA514520 / Invitrogen #13-2444-82 / BD Pharmingen, # BD553076: validated by manufacturer for Western blot, immunohistochemistry (paraffin and frozen), immunocytochemistry, and flow cytometry.

Animals and other research organisms

Policy information about studies involving animals; ARRIVE guidelines recommended for reporting animal research, and Sex and Gender in Research

Laboratory animals	Male and female BL6 mice at 6 weeks of age; male immunocompromised NOD/Shi-scid, IL-2Rγnull (NOG) mice at 6 weeks of age. Mice were maintained at the appropriate biosafety level under constant temperature and humidity conditions with a 12h light cycle. Animals were allowed food and water ad libitum.

Wild animals	No wild animals were used in this study.
Reporting on sex	Male and female mice were used according to previously published models.
Field-collected samples	No field-collected samples were used in this study.
Ethics oversight	Smac mimetic mouse model experiments were approved by the local ethics committee (WEHI AEC# 2022.009). Humanized mouse model experiments were approved by the local ethics committee and the institutional review board of Niigata University.

Note that full information on the approval of the study protocol must also be provided in the manuscript.

Clinical data

Policy information about clinical studies

All manuscripts should comply with the ICMJE guidelines for publication of clinical research and a completed CONSORT checklist must be included with all submissions.

Clinical trial registration	n/a
Study protocol	n/a
Data collection	Tissue biopsies represent the original biopsies used for diagnostic purposes during clinical routine.
Outcomes	n/a
