# Supplementary material for: Spatial proteomics identifies JAKi as treatment for a lethal skin disease
Source: Nature. 2024 Oct 16;635(8040):1001–9. doi: 10.1038/s41586-024-08061-0 (PMC11602713; doi:10.1038/s41586-024-08061-0)
Supplement: Supplementary file 3 — Supplementary Tables 1–14 [file 41586_2024_8061_MOESM3_ESM.zip › 2023-09-16426F-s3/2023-09-16426F-Supplementary Information Guide.docx]

Supplementary Table 1

Anonymized baseline characteristics of DVP cohort

Corresponds to: Fig. 1-2, 4a-b; Extended Data Fig. 1, 3b-c, 4a-b, 4c

Supplementary Table 2

Anonymized baseline characteristics of mDIA-DVP cohort

Corresponds to: Fig. 3; Extended Data Fig. 3a

Supplementary Table 3

Anonymized baseline characteristics of Nanostring cohort

Corresponds to: Fig. 4c-d; Extended Data Fig. 5

Supplementary Table 4

Anonymized baseline characteristics of Phosphoproteomic cohort

Corresponds to: Extended Data Fig. 6

Supplementary Table 5

Anonymized baseline characteristics of patients treated with JAK inhibitors

Corresponds to: Fig. 6; Extended Data Fig. 9 - 10.

Supplementary Table 6

Antibody information

Supplementary Table 7

Processed raw data of lesional keratinocytes (DVP)

Supplementary Table 8

Processed raw data of lesional immune cells (DVP)

Supplementary Table 9

Log2 normalised proteome of CD4, CD8, CD163 and healthy(CD45) immune cells (mDIA_DVP)

Supplementary Table 10

Log2 normalised proteome of detached (TEN), attached (TEN) and control (healthy) keratinocytes (mDIA_DVP)

Supplementary Table 11

Log2 normalised, imputed class-I phosphoproteome and metadata

Supplementary Table 12

Normalised count matrix (Nanostring)

Supplementary Table 13

Clinical scores (D1,D3), average dermal thickness, lesion size, % weight change measurements (smac-mimetic mouse model)

Supplementary Table 14

TUNEL quantification (humanised mouse model)
